# Integrative multiomics reveals common endotypes across *PSEN1*, *PSEN2*, and *APP* mutations in familial Alzheimer’s disease

**DOI:** 10.1186/s13195-024-01659-6

**Published:** 2025-01-04

**Authors:** Phoebe Valdes, Andrew B. Caldwell, Qing Liu, Michael Q. Fitzgerald, Srinivasan Ramachandran, Celeste M. Karch, Dominantly Inherited Alzheimer Network (DIAN), Celeste M. Karch, Sarah Adams, Ricardo Allegri, Aki Araki, Nicolas Barthelemy, Randall Bateman, Jacob Bechara, Tammie Benzinger, Sarah Berman, Courtney Bodge, Susan Brandon, William Bill Brooks, Jared Brosch, Jill Buck, Virginia Buckles, Kathleen Carter, Lisa Cash, Charlie Chen, Jasmeer Chhatwal, Patricio Chrem Mendez, Jasmin Chua, Helena Chui, Laura Courtney, Carlos Cruchaga, Gregory S. Day, Chrismary DeLaCruz, Darcy Denner, Anna Diffenbacher, Aylin Dincer, Tamara Donahue, Jane Douglas, Duc Duong, Noelia Egido, Bianca Esposito, Anne Fagan, Marty Farlow, Becca Feldman, Colleen Fitzpatrick, Shaney Flores, Nick Fox, Erin Franklin, Nelly Joseph-Mathurin, Hisako Fujii, Samantha Gardener, Bernardino Ghetti, Alison Goate, Sarah Goldberg, Jill Goldman, Alyssa Gonzalez, Brian Gordon, Susanne Gräber-Sultan, Neill Graff-Radford, Morgan Graham, Julia Gray, Emily Gremminger, Miguel Grilo, Alex Groves, Christian Haass, Lisa Häsler, Jason Hassenstab, Cortaiga Hellm, Elizabeth Herries, Laura Hoechst-Swisher, Anna Hofmann, David Holtzman, Russ Hornbeck, Yakushev Igor, Ryoko Ihara, Takeshi Ikeuchi, Snezana Ikonomovic, Kenji Ishii, Clifford Jack, Gina Jerome, Erik Johnson, Mathias Jucker, Stephan Käser, Kensaku Kasuga, Sarah Keefe, William Klunk, Robert Koeppe, Deb Koudelis, Elke Kuder-Buletta, Christoph Laske, Allan Levey, Johannes Levin, Yan Li, Oscar Lopez, Jacob Marsh, Ralph Martins, Neal Scott Mason, Colin Masters, Kwasi Mawuenyega, Austin McCullough, Eric McDade, Arlene Mejia, Estrella Morenas-Rodriguez, John Morris, James Mountz, Cath Mummery, Neelesh Nadkarni, Akemi Nagamatsu, Katie Neimeyer, Yoshiki Niimi, James Noble, Joanne Norton, Brigitte Nuscher, Ulricke Obermüller, Antoinette O’Connor, Riddhi Patira, Richard Perrin, Lingyan Ping, Oliver Preische, Alan Renton, John Ringman, Stephen Salloway, Peter Schofield, Michio Senda, Nicholas T. Seyfried, Kristine Shady, Hiroyuki Shimada, Wendy Sigurdson, Jennifer Smith, Lori Smith, Beth Snitz, Hamid Sohrabi, Sochenda Stephens, Kevin Taddei, Sarah Thompson, Jonathan Vöglein, Peter Wang, Qing Wang, Elise Weamer, Chengjie Xiong, Jinbin Xu, Xiong Xu, Douglas R. Galasko, Shauna H. Yuan, Steven L. Wagner, Shankar Subramaniam

**Affiliations:** 1 Department of Bioengineering, University of California, San Diego, La Jolla, CA 92093 USA; 2 Bioengineering Graduate Program, University of California, San Diego, La Jolla, CA 92093 USA; 3 Department of Neurosciences, University of California, San Diego, La Jolla, CA 92093 USA; 4 VA San Diego Healthcare System, San Diego, CA 92161 USA; 5 Department of Psychiatry, Washington University in St. Louis School of Medicine, St. Louis, MO 63110 USA; 6 Department of Cellular and Molecular Medicine, University of California, San Diego, La Jolla, CA 92093 USA; 7 Department of Nanoengineering, University of California, San Diego, La Jolla, CA 92093 USA; 8 Department of Computer Science and Engineering, University of California, San Diego, La Jolla, CA 92093 USA; 9 Present Address: Department of Obstetrics, Gynecology, and Reproductive Sciences, University of California, San Diego, La Jolla, CA 92093 USA; 10 Present Address: N. Bud Grossman Center for Memory Research and Care, Department of Neurology, University of Minnesota, GRECC, Minneapolis VA Health Care System, Minneapolis, MN 55417 USA

## Abstract

**Background:**

*PSEN1, PSEN2,* and *APP* mutations cause Alzheimer’s disease (AD) with an early age at onset (AAO) and progressive cognitive decline. *PSEN1* mutations are more common and generally have an earlier AAO; however, certain *PSEN1* mutations cause a later AAO, similar to those observed in *PSEN2* and *APP*.

**Methods:**

We examined whether common disease endotypes exist across these mutations with a later AAO (~ 55 years) using hiPSC-derived neurons from familial Alzheimer’s disease (FAD) patients harboring mutations in *PSEN1*^*A79V*^, *PSEN2*^*N141I*^*, and APP*^*V717I*^ and mechanistically characterized by integrating RNA-seq and ATAC-seq.

**Results:**

We identified common disease endotypes, such as dedifferentiation, dysregulation of synaptic signaling, repression of mitochondrial function and metabolism, and inflammation. We ascertained the master transcriptional regulators associated with these endotypes, including REST, ASCL1, and ZIC family members (activation), and NRF1 (repression).

**Conclusions:**

FAD mutations share common regulatory changes within endotypes with varying severity, resulting in reversion to a less-differentiated state. The regulatory mechanisms described offer potential targets for therapeutic interventions.

**Supplementary Information:**

The online version contains supplementary material available at 10.1186/s13195-024-01659-6.

## Introduction

Familial Alzheimer’s disease (FAD) is an inherited neurodegenerative disorder caused by autosomal-dominant mutations in the presenilin-1 (*PSEN1*), presenilin-2 (*PSEN2*), and amyloid-β precursor protein (*APP*) genes [1]. FAD patients demonstrate the hallmark progressive memory loss and cognitive decline as the more common sporadic Alzheimer’s disease (SAD), albeit with age at onset (AAO) occurring substantially earlier (~ 30–62 years) [2]. There have been over 300 mutations identified across these three genes, the majority of which cause autosomal dominant AD; mutations in *PSEN1* are the most prevalent (~ 80%), followed by *APP* (~ 14%) and *PSEN2* (~ 5%) [3]. The majority (72%) of variation in disease AAO in *PSEN1* can be explained by mutation alone. However, a spectrum exists in median survival within mutations [2, 4]. In contrast, mutations in *PSEN2* demonstrate more variable penetrance than *PSEN1* or *APP * [5]. Generally, mutations in *PSEN1* cause particularly early AAO, with symptoms occurring as early as 30 years for mutations like *PSEN1*^*G206V*^ [2, 6]. In contrast, mutations in *APP* and *PSEN2* lead to a markedly later AAO (~ 50–60 years). These three genes share a functional molecular link: both *PSEN1* and *PSEN2* constitute the catalytic component within the gamma-secretase complex, which carries out the intra-membrane cleavage at the γ-cleavage site in the sequential proteolytic processing of APP into amyloid β (Aβ) peptides [7]. The aberrant production of longer Aβ peptides, particularly Aβ42, leads to plaque formation, a hallmark of AD pathology. Although FAD is exceedingly rare (~ 1% of all AD cases), these mutations have contributed substantially to understanding AD pathogenesis. FAD is hypothesized to represent an accelerated form of the more common sporadic AD since both share the same pathological (Aβ plaques, tau tangles, synapse and neuron loss) and clinical features (progressive memory loss, cognitive decline, and death). Whether these pathological features describe the etiology of any AD is a matter of debate, particularly with the recently documented failures of Aβ-targeting therapeutics to stop or slow cognitive decline. Regardless of whether these Aβ plaques initiate disease onset, FAD mutations lead to differential levels of Aβ peptides. Moreover, there is evidence that the ratio of shorter Aβ peptides (e.g., Aβ38, Aβ40) to longer forms (e.g., Aβ42, Aβ43) correlates with disease AAO in FAD [8–10]. Beyond the alteration of Aβ peptide levels, the pathophysiological functions of *APP*, *PSEN1*, and *PSEN2* remain inconclusive. The gamma-secretase complex has numerous substrates beyond APP, including Notch, N-cadherin, E-cadherin, ErbB4, and CD44 [11]. It has been shown to regulate the turnover and activity of other signaling pathways, including Wnt and EGFR [12, 13]. Furthermore, mutations in *PSEN1* (and to a lesser extent in *PSEN2*) may affect the endoproteolytic activity of the gamma-secretase complex, potentially modifying the overall activity and half-life of the enzyme [12]. A monumental challenge in AD research has been the inability to study disease progression in any way other than invasive approaches. Although -omics characterization of postmortem patient brains has revealed the dysregulation that occurs over the course of AD, these types of analyses offer a snapshot of the late- or end-stage of the disease. Further, there is limited postmortem patient brain sequencing data available for familial AD, as the vast majority of available postmortem data comes from sporadic AD, which is highly heterogeneous relative to the familial form. In contrast, patient-derived brain cells, either through iPSC differentiation or direct conversion, circumvent the limitations of the postmortem brain and allow for interrogation of early AD mechanisms in human, disease-relevant models. Previous work by us and others have used transcriptomic and chromatin profiling in patient-derived neurons to identify disease endotypes [14], which are pathobiological mechanisms that underlie or encompass Alzheimer’s disease phenotypes, including repression of neuronal lineage and activation of dedifferentiation to alternative lineages, in both FAD and SAD [15–17]. While the availability of iPSC-derived familial AD patient lines is limited, we have demonstrated that the dysregulation of these specific cellular functions is also mirrored in the postmortem brains of patients harboring *PSEN1* mutations [15, 18, 19]. While these endotypes were commonly observed across diverse *PSEN1* mutations [15], and the endpoint phenotypes (i.e., histopathological and clinical) are shared among *PSEN1*, *PSEN2*, and *APP* mutations, it is unclear whether mutations in *PSEN2* and *APP* cause the same dysregulation within disease-associated gene regulatory programs. Although *PSEN1* mutations tend to have the earliest AAO amongst the three FAD genes, *PSEN1* mutations with a later AAO comparable to *PSEN2* and *APP* mutations also exist*.* Therefore, we aimed to characterize FAD patient-derived neurons by RNA-seq and ATAC-seq to identify common and unique endotypes associated with prevalent mutations [20, 21] from each FAD gene with a similar AAO (*PSEN1*^*A79V*^, ~ 55–60 years [9, 22]; *PSEN2*^*N141I*^, ~ 54 years [21]; *APP*^*V717I*^, ~ 55–57 years [23, 24]). Here, we demonstrate that while the *PSEN1*^*A79V*^ mutation exhibits similar levels of endotype modulation observed in other *PSEN1* mutations [15], the *APP*^*V717I*^ mutation shows subtle modulation of the same endotypes with the same direction of change; in contrast, the *PSEN2*^*N141I*^ mutation has a similar magnitude of endotype modulation albeit sometimes occurring in the opposite direction.

## Methods

### hiPSC neuron generation

Fibroblasts were derived from patient skin biopsies from adult human volunteers; Non-demented control (NDC) and *APP*^*V717I*^ were generated at the Alzheimer’s Disease Research Center at the University of California, San Diego (UCSD) per UC San Diego IRB approval [15, 25, 26], whereas *PSEN1*^*A79V*^ and *PSEN2*^*N141I*^ were generated at the Dominantly Inherited Alzheimer’s Network (DIAN) at Washington University per the Washington University School of Medicine Institutional Review Board and Ethics Committee (IRB 201104178, 201,306,108) [27]. *PSEN1*^*A79V*^ and *PSEN2*^*N141I*^ fibroblasts were transformed with retroviral transduction using *OCT3/4*, *KLF4*, *SOX2* and *c-MYC * [26, 27]. NDC and *APP*^*V717I*^ fibroblasts were transformed using the episcopal method to introduce reprogramming factors [28, 29]. Neurons were differentiated from iPSCs as previously described [15, 29, 30]; PA6 cells were plated in a 10 cm dish and seeded with 100,000 iPSCs the next day. To enhance neural induction, cultures were treated with 5 μM dorsomorphin (Sigma) and 10 μM SB431542 (Tocris) for the first 6 days of differentiation. On day 12, neural stem cells (NSCs) were sorted using cell surface signature CD24^+^/CD184^+^/CD44^−^/CD271^−^ and seeded at 150 K/cm^2^ on a plastic dish coated with Matrigel (83 μg/ml). NSCs were expanded in NSC growth medium (DMEM:F12 + Glutamax™ (Thermo Fisher Cat. 10,565,018), 1 × B-27 (Thermo Fisher Cat. 17,504,044), 1 × N-2 (Thermo Fisher Cat. 17,502,001), 1 × Penicillin–Streptomycin (Thermo Fisher Cat. 15,070,063), and 20 ng/mL human bFGF-2 (BioPioneer Cat. HRP-0011)). At 80% confluence, the medium was changed to neuron differentiation medium (DMEM:F12 + Glutamax™, 1 × B-27, 1 × N-2, 1 × Penicillin–Streptomycin) for 3 weeks of differentiation followed by culture dissociation with Accutase (Sigma Cat. A6964) [15, 29, 31]. Cells were resuspended in 200 μL of iMag buffer (1 × neural differentiation medium, 0.5 µM EDTA, 0.5% Bovine Serum Albumin) and incubated with PE Mouse Anti-Human CD184 and CD44 antibodies (BD Biosciences Cat. 561,733 and 561,858, respectively) for 15 min on ice in the dark. The mixture was washed with iMag buffer and subsequently incubated with anti-PE conjugated magnetic beads (BD Biosciences) for 30 min at room temperature as described [15, 29, 32]. Magnetic bead separation was carried out for 8 min according to the manufacturer’s protocol (BD Biosciences). The supernatant containing purified CD184^−^/CD44^−^ neurons were removed and spun down for downstream applications.

### RNA-seq and data processing

Total RNA from magnetically purified human NDC, *PSEN1*^*A79V*^, *PSEN2*^*N141I*^, and *APP*^*V717I*^ hiPSC-derived neurons (*n* = 3 replicates differentiated in parallel from individual donor patients) using the RNeasy Plus Micro Kit (QIAGEN, catalog no. 74034) according to the manufacturer’s protocol. On-column deoxyribonuclease digestion was performed on total RNA extracts to remove any genomic contamination (QIAGEN, catalog no 79254). Libraries were prepared for RNA-seq using the TruSeq Stranded Total RNA Library Prep Kit (Illumina, catalog no. RS-122–2303) by the Ribo-Zero ribosomal RNA reduction method (Illumina, catalog no. MRZG12324). Samples were sequenced at the UC San Diego Institute for Genomics Medicine (IGM) sequencing core on an Illumina HiSeq 4000 generating paired-end, 100-bp reads with an average of 25 million reads per sample (Illumina, catalog no. FC-410–1001). Preprocessing of RNA-seq data was conducted using the TrimGalore! Package v0.6.4, removing adapter sequences and low-quality reads using CutAdapt v1.18 [33]. Trimmed RNA-Seq reads were mapped to the GRCh38.99 human transcriptome using kallisto v0.46.1 [34] with the options -bias -rf-stranded -b 100 followed by transcript level summation to the gene level using the R package *tximport* v1.18.0 [35]. Lowly expressed genes were filtered out using *filterByExpr,* and counts were normalized using the weighted mean trimmed of M-values (TMM) in the R package *edgeR* v3.32.1 [36]. Normalized, filtered counts were used for differential gene expression (DGE) analysis of all three FAD mutations relative to the NDC control samples using the *voomwithQualityWeights * [37] function within the *limma* v3.46.0 [38] R package. Differentially expressed genes (DEGs) from the filtered gene list of 22,310 genes were defined using a false discovery rate (FDR)-adjusted *p*-value (*p*) cutoff of < 0.05 using the Benjamini–Hochberg correction method from the *eBayes* differential t-test. Quasi-proportional Venn diagrams of DEG overlap between the FAD mutations were generated using the *nVennR* v0.2.3 package in R [39]. Rank Rank Hypergeometric Overlap (RRHO) analysis between the three mutation pairs was performed using the *RRHO2* package in R [40, 41]. For reanalysis of *PSEN1ΔE9* and NDC iPSC-derived astrocytes published previously [42], RNA-seq files were downloaded from the GEO series GSE138695 and processed using the same pipeline described above for iPSC-derived neuron RNA-seq analysis.

### Neuron cell type marker analysis

To validate the differentiation of NDC and FAD mutation patient lines into neurons across the two different iPSC reprogramming methods, we combined the NDC and FAD neuron RNA-seq data collected here with two additional mutations (*PSEN1*^*H163R*^ and *PSEN1*^*A431E*^) we published previously [15]. We identified markers for different subtypes of mature neurons (i.e., GABAergic, dopaminergic, and glutamatergic), general markers for synaptic, pan-neuronal, and amyloid-associated genes, and markers for six cortical layer groups. Markers were selected from neuronal cell identity marker resources and published literature sources for GABAergic [43–49], dopaminergic [50–55], glutamatergic [44, 56–58], synaptic [43, 48], pan-neuronal [59, 60], amyloid-associated, and cortical layer [61, 62] neuron types. Violin plots for RNA expression values were generated in GraphPad Prism v10.1. For z-score heatmaps, filtered, normalized gene counts were z-scored using the *scale* function from the *base* v4.2.3 R package with the parameters set at center = TRUE and scale = TRUE and plotted with the *heatmap* function from the *stats* R package. Neuron identity markers differentially expressed using the *topTable* function from the *limma* [38] R package with a FDR *p*-value cutoff < 0.05 were marked with an asterisk.

### tSNE Clustering analysis

To perform clustering analyses for RNA-seq, t-distributed Stochastic Neighbor Embedding (tSNE) [63] using a Barnes-Hut Implementation [64] was performed using the *Rtsne * [65] R package. Filtered log_2_ normalized count data for key genes involved in five disease-relevant endotypes [66] were used to perform unsupervised tSNE clustering. The following parameters were used to implement *Rtsne*: pca = TRUE; perplexity = 5 (defining a small number of loose neighbors for each sample point); theta = 0.1; truncated partial_pca = TRUE (PCA to calculate principal components); initial_dims = 17 (retain PCA dimensions); max_iter = 5000; pca_center = TRUE, pca_scale = TRUE.

### TF activity analysis

Transcription factor (TF) activity was assessed with ISMARA [67] and DoRothEA [68]. For ISMARA analysis, quality and adaptor trimmed fastq.gz RNA-seq data files for all hiPSC-derived neurons were uploaded to the ISMARA server (ismara.unibas.ch) for processing and sample averaging. The directional z-score for each enriched TF motif was calculated by multiplying the sign of the Pearson correlation (i.e., the direction of change) between each motif and its target genes with each z-score of the TF motif (FAD vs. NDC) and the direction of change in expression for said target genes (i.e., -1 for downregulated genes, + 1 for upregulated genes). For DoRothEA analysis, the gene ranking for each FAD mutation relative to NDC was calculated by applying quantile normalization to the *limma* FDR-adjusted *p*-value using the Benjamini–Hochberg correction method divided by two multiplied by the sign of the log_2_FC for each gene in the comparison. TF activity was then calculated using the *msviper* function in the *viper * [69] R package with the DoRothEA C regulon.

### Gene set enrichment and pathway analysis

Gene set enrichment and pathway analysis for RNA-seq of individual FAD mutations was performed using two approaches: 1) the *fgseamultilevel* function in the *fgsea * [70] R package and 2) the *tmodCERNOTest* function in the *tmod * [71] R package coupled with the GOBP [72] and Hallmark [73] databases for pathway and ontology enrichment or the ENCODE-ChEA [74] and ReMap [75] databases for TF-gene target enrichment*.* Genes were ranked by the *limma* *t*-value for the *fgsea* statistical enrichment test, whereas genes were ranked by minimum significant distance (msd) for the *CERNO * [76] test. For simultaneous enrichment of all FAD mutations, we performed rank-MANOVA enrichment using the *mitch * [77] R package on confects-ranked gene lists generated with the *topconfects* [78] R package (0.05 FDR-adjusted *p*-value for confidence interval calculations).

### Differential co-expression modular network analysis

Co-expression gene modules discovery across all FAD mutations and NDC was performed using the *cemitool* function in the *CEMiTool * [79] R package using the following parameters: apply_vst = TRUE; filter = TRUE; filter_pval = 0.05; network_type = ”signed”. Following module identification, the differential activity change of each co-expression gene module in FAD mutations relative to NDC was performed using the *fgseamultilevel* function in the *fgsea * [70] R package.

### Combined TF-gene regulatory and PPI network construction

Regulatory interaction networks for co-expression gene modules 1, 3 and 4 were constructed using high-confidence, protein–protein interactions (PPI) edges from the STRING (11.0) database [80] and TF-gene regulatory edges from the ENCODE-ChEA and ReMap databases. For StringDB PPI edges, we defined high-confidence interactions as those with both experimental and database evidence, with a composite score of the two interaction sources > 400. For a given module interaction network, module genes were included as source and target nodes. In contrast, first neighbor genes (i.e., genes not in the module that have a StringDBv11 PPI interaction with module genes) were included as target nodes. Hypergeometric enrichment of module and first neighbor genes (i.e., genes that are not in a module but share a StringDB PPI edge with a module gene) was performed using the *tmodHGtest* function in the *tmod *R package with the GOBP and Hallmark geneset databases and ENCODE-ChEA and ReMap TF-gene target databases. Key TFs were selected based on hypergeometric enrichment of module and first neighbor genes using the ENCODE-ChEA and ReMap TF-gene target databases. Network images were generated in Cytoscape v3.8.2 [81].

### ATAC-seq and data processing

ATAC-seq transposition experiments were performed as previously described [15, 82] on 50,000 cells in NDC, *PSEN1*^*A79V*^, *PSEN2*^*N141I*^, and *APP*^*V717I*^ hiPSC-derived neurons (replicates, *n* = 3) using the Illumina Nextera DNA Sample Preparation Kit (Illumina, catalog no. 15028523) and the QIAGEN MinElute PCR Purification Kit (QIAGEN, catalog no. 28004). ATAC-seq libraries were generated from transposed DNA using the Kapa Biosystems Real-Time Library Amplification Kit (Kapa Biosystems, catalog no. 07959028001) as recommended by the manufacturer, monitoring amplification by qPCR and stopping the reaction when all samples reached a fluorescence amplification intensity between standards 1 and 3. ATAC-seq libraries were then further purified using the QIAGEN MinElute PCR Purification Kit and sequenced at the UC San Diego IGM sequencing core on an Illumina HiSeq 4000 platform generating paired-end, 50-bp reads with an average of 25 million reads per sample. ATAC**-**seq data preprocessing was performed using TrimGalore! to remove sequencing adaptors and low-quality reads. Trimmed reads were then aligned to the GRCh38 human genome (GCA_000001405.15 with no alternative analysis) using BBMap v37.95 in the BBTools [83] suite with the options maxindel = 20 ambig = random, followed by sorting and indexing of bam files using SAMtools v1.9 [84], and annotation of PCR duplicates using the Picard v2.23.3 MarkDuplicates function with the option VALIDATION_STRINGENCY = LENIENT. All duplicates and mitochondrial, chromosome X, chromosome Y, and EBV reads were removed using SAMtools v1.9 command view with the options -b -h -f 3 -F 4 -F 8 -F 256 -F 1024 -F 2048. To determine open chromatin regions, HMMRATAC v1.2.5 [85] was used to call peaks on the ATAC-seq data and determine open chromatin regions with the options –m 50,200,400,600 –score all. These open chromatin regions were then passed to the *Diffbind* R package v3.0.15 [86] to determine regions of differential accessibility between NDC and each FAD mutant condition. Consensus peaks selection for each condition was identified (minimum overlap = 3) and the subsequent peaksets for all conditions were merged using the *dba.peakset* function. Read coverage over the combined consensus peakset was determined using the *dba.count* function with the DBA_SCORE_TMM_READS_EFFECTIVE peak scoring option. Differentially accessible peaks for each FAD condition relative to NDC were determined with *the* edgeR method with a FDR-adjusted *p*-value of < 0.05 using the Benjamini–Hochberg correction method and then subsequently annotated using the *annotatePeak* function in the *ChIPseeker* R package v1.26.2 [87], defining the promoter region − 1500 to 500 bp from the TSS. Enhancer-associated ATAC-seq regions were defined as differential peaks occurring within the PEREGRINE enhancer region list [88] and then finding the intersecting non-promoter based DNA regions with the *join_overlap_inner* function in the *plyranges* R package [89].

### ATAC-seq TF activity and enrichment analysis

To assess TF activity associated with differential chromatin accessibility, we performed HINT-ATAC for differential TF footprinting and GimmeMotifs *maelstrom* for differential motif activity. Using the consensus ATAC-seq peakset, HINT v.0.13.1 [90, 91] was run with the parameters: rgt-hint function *footprinting*, options –atac-seq –paired-end –organism = hg38 to identify TF footprints in each sample; rgt-motif analysis function *matching* to match footprints to known TFs in the Catalog of Inferred Sequence Binding Preferences (cisBP) v2.00 [92] human motif database; and rgt-hint function *differential*, options –bc –nc –window-size 200 –standardize. GimmeMotifs [93, 94]* maelstrom* was run using the Swiss Regulon [95] human pwm motif database with the default parameters following read quantification, log-transformation, and mean-centering per row for all FAD and NDC neuron ATAC-seq for the consensus peakset. To determine enriched pathways and ontologies for differential ATAC-seq regions, we used the *chiprenrich* logistic regression model test function in the *chipenrich* R package [96] using the ENCODE-ChEA and ReMap TF-gene target databases and GOBP and Hallmark ontology databases. For promoter-associated peaks with increased or decreased accessibility for a given FAD vs. NDC comparison, the locus definition *nearest_tss* was used; for enhancer-associated peaks, we generated a custom locus definition map using the PEREGRINE enhancer database to match putative differential ATAC-seq enhancer peaks to the corresponding gene in PERGERINE. Promoter and enhancer ATAC-seq coverage plots were generated with the Deeptools [97] v3.5.0 functions *computeMatrix* and *plotHeatmap*.

### ATAC-seq GWAS loci analysis

We sought to assess whether known AD-associated genome-wide association study (GWAS) loci variants or GWAS-identified single nucleotide polymorphisms (SNPs) occur within FAD differentially accessible regions (DARs). To carry this out, we intersected DARs from each mutation with a collection of genetic variants based on unique rsID information associated with AD risk from literature sources (*n* = 2,644 total variants) using the *join_overlap_inner* function in the *plyranges* v1.14.0 R package. These sources spanned from the following: 1) > 200 GWAS publications from the Alzheimer’s disease Genetics Consortium and other consortia collectively grouped in a publicly available database called the Alzheimer’s Disease Variant Portal (ADVP) (*n* = 1,821 variants) [98], 2) 111,326 clinically diagnosed/’proxy’ AD and 677,663 controls involved in a two-stage GWAS study, as part of the European Alzheimer & Dementia biobank (EADB) and the Trans-Omics for Precision Medicine (TOPMed) databases (*n* = 271 variants) [99] and 3) polygenic risk score (PRS) extreme group classification from AD individual data as part of the UK Biobank database (*n* = 552 variants) [100]. Variants with a reported association *p*-value > 0.05, as observed in the different GWAS studies, were removed. Quasi-proportional Venn diagrams of DAR-variant overlap across the FAD mutations were generated using the *nVennR* v0.2.3 package in R [39]. ATAC-seq gene locus coverage plots with SNP genetic variants were generated in IGV [101].

### Integrated analysis of RNA-seq and ATAC-seq data

To find genes with differential chromatin accessibility and corresponding differential gene expression, we took the intersect of Ensembl genes with differential gene expression and an associated peak with differential chromatin accessibility passing a threshold of FDR *p*-value < 0.05. Next, we used the *limma_confects* and *edgeR_confects* functions in the *topConfects* R package to calculate the confident effect size for each gene and ATAC-seq peak, respectively. For genes with multiple differential peaks, we prioritized the peaks located in promoters and abs(confect). For all peaks associated with genes identified by RNA-seq, we calculated a z-score for the confect score for ATAC-seq and RNA-seq and multiplied the two z-scores (presented in log_2_ space) to generate a correlation score of each ATAC-seq peak differential chromatin accessibility with the corresponding gene’s RNA-seq expression level. For TF-gene target and ontological enrichment following integration, we performed a hypergeometric test using the *tmodHGtest* function in the *tmod * R package with the ENCODE-ChEA and ReMap TF databases and the GOBP and Hallmark databases. A defined background list of all genes with differentially accessible ATAC-seq peaks in each condition was used for the *tmod* hypergeometric test. In order to estimate differential TF activity, we used diffTF [102] to classify TFs into either repressors or activators after integrating chromatin accessibility data (ATAC-seq) with gene expression data (RNA-seq). The cisBP TF binding site (TFBS) [92] database with 923 motifs was used with the following parameters: maxCoresPerRule: 2; dir_TFBS_sorted: true; regionExtension: 100; designContrast: ~ conditionSummary; designVariableTypes: conditionSummary:factor; nPermutations: 0; nBootstraps: 1000; nCGBins: 10; and RNASeqIntegration: true in order to identify the differential activity of TFs for each mutation relative to NDC (with a significance cutoff of FDR *p*-value < 0.05). To estimate the ontologies and TFs whose targets have the highest correlation between differential accessibility (in promoter and enhancer regions) and differential gene expression, we used the *intePareto * [103] R package and CERNO ranked enrichment. We first used the *bam2counts* function to calculate the read density for each condition over all PEREGRINE enhancer-associated regions and used the *doMatch* function to calculate the read density for each condition for promoter regions. Z-scores for enhancer-gene and promoter-gene were calculated with the *doIntegration* function, and pareto optimization was performed with the *doPareto* function. After generating a pareto-optimized gene ranking for the integrated ATAC-seq and RNA-seq data, we used this ranked gene list as an input for CERNO enrichment in the *tmod* R package using the *tmodCERNOtest* function with the ENCODE-ChEA, ReMap, and a custom neural-specific TF regulon database we previously generated [15] for TF target enrichment and the GOBP and Hallmark databases for ontology and pathway enrichment. All ATAC-seq gene locus coverage plots were generated in IGV [101]. Code for all sequencing analysis is available at 10.5281/zenodo.8267332.

### Integrated drug target analysis of RNA-seq and ATAC-seq data

To perform the identification of drug pathway targets in integrated RNA-seq and ATAC-seq data, we took drug agents from different clinical trial phases (Phases 1, 2, and 3) for treatment of Alzheimer’s disease associated with the Common Alzheimer’s Disease Research Ontology (CADRO) mechanism classes (*n* = 154) [104], and intersected those with *intePareto*-ranked enriched pathways from CERNO, manually curated with CADRO-based classes. Pie chart distribution of drug agents associated with CADRO-based enriched integrated pathways common among all FAD mutations were created using GraphPad Prism v9.5.0 software. Quasi-proportional Venn diagrams of drug agent overlap between the FAD mutations were generated using the *nVennR* v0.2.3 R package [39] for the different drug phase trials. In addition, we calculated the overlap of enriched pathways across all FAD neurons with shared drug agents using the *chordDiagram* function. For identifying the predicted drug targets based on genes from integrated RNA-seq and ATAC-seq data, we performed the intersection of genes in each FAD mutation with both differential gene expression and differential ATAC peaks (from chromatin-accessible promoter and PEREGRINE-enhancer regions). These DEG/DAR genes were intersected with 1,608 FAD-approved drug targets, 2,251 unique human target notes, and high-quality 15,367 physical drug-target interactions (edges) as part of a drug-target network constructed using published binding affinity data [105] that is publicly available in a systems biology tool known as AlzGPS [106]. Quasi-proportional Venn diagrams of predicted drug target overlap between the FAD mutations were generated using the *nVennR* v0.2.3 R package [39]. Finally, we calculated the overlap of drug target DEGs in FAD neurons with shared FDA-approved drugs using the *chordDiagram* function in the *circlize* v0.4.15 R package [107].

## Results

**Fig. 1:**
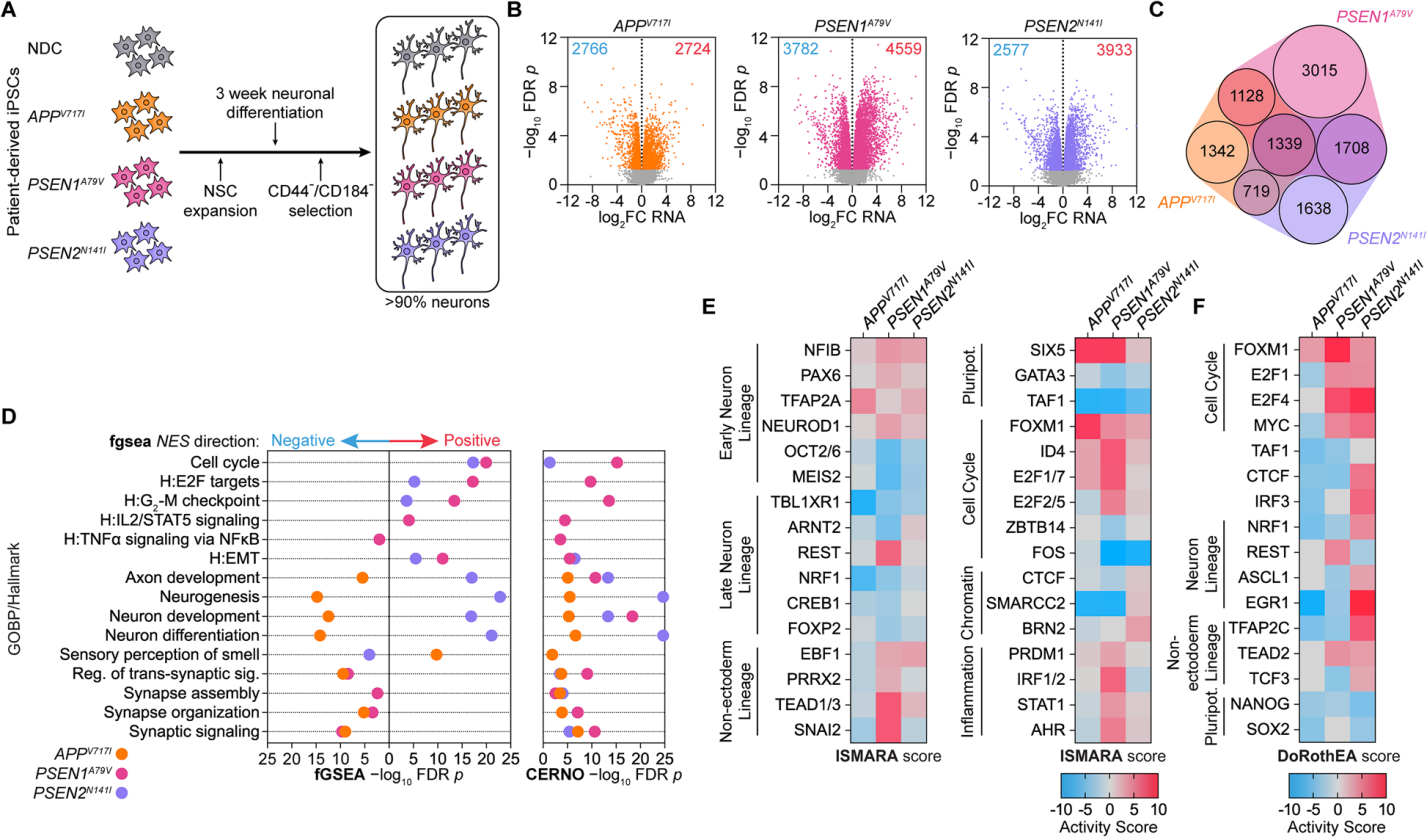
Transcriptomic profiling of FAD hiPSC-derived neurons*.* **A** Patient-derived Non-Demented Control (NDC), *APP*^*V717I*^, *PSEN1*^*A79V*^, and *PSEN2*^*N141I*^ hiPSCs were differentiated into neurons and purified by CD44^−^/CD184.^−^ selection. **B** RNA-Seq volcano plots of differentially expressed genes (DEGs) across the three FAD mutations relative to non-demented control (NDC) as determined by *limma* with an FDR *p*-value (*p*) < 0.05. **C** Quasi-proportional Venn diagram overlap of DEGs across the three FAD mutant hiPSC-derived neurons. **D** Gene Ontology: Biological Process (GOBP) and Hallmark database geneset enrichment using the *fgsea* multilevel enrichment test (left) or *tmod* CERNO enrichment test (right); dot plots indicate significant (-log_10_ FDR *p*-value < 0.05) pathways in each mutation relative to NDC. **E**–**F** Common Transcription Factors (TFs) across the FAD mutations with predicted significant activity change by (**E**) ISMARA motif analysis (based on z-score, TF-gene Pearson correlation, and average gene target expression change) or (**F**) *DoRothEA* TF-gene target analysis (Normalized Enrichment Score)

Non-demented control (NDC), *PSEN1*^*A79V*^, *PSEN2*^*N141I*^, and *APP*^*V717I*^ hiPSCs were differentiated into CD44^−^/CD184^−^ neurons as previously described [15, 29] (Fig. 1A; Supplementary Fig. 1A-B). RNA-seq and subsequent differential gene expression analysis identified a substantial number of differentially expressed genes (DEGs) relative to NDC in all three mutations (Fig. 1B), with 1339 common DEGs (Fig. 1C). Rank Rank Hypergeometric Overlap (RRHO) analysis revealed the strongest similarity overlap between the *PSEN1*^*A79V*^ and *PSEN2*^*N141I*^ mutations and the weakest similarity overlap between the *APP*^*V717I*^ and *PSEN2*^*N141I*^ mutations (Figure S1C). To assess the consistency of neuron differentiation across all NDC and FAD patient lines, we investigated the expression of key marker genes for specific neuronal subtypes (GABAergic, dopaminergic, and glutamatergic; Supplementary Fig. 2A-D), broad neuronal cells (synaptic, pan-neuronal, and Amyloid-associated; Supplementary Fig. 3A-D), and cortical layers (layers 1–6; Supplementary Fig. 4A-H). This showed that most of key markers for each neuron subtype or aspect are present in all FAD mutations and control lines, particularly observing consistent expression of pan-neuronal markers *ENO2*, *RBFOX3* and *MAP2* (Supplementary Fig. 3C). Further, we observed greater differential expression correlation by RRHO between mutations in the same FAD gene but with different iPSC reprogramming methods (*PSEN1*^*A79V*^ and *PSEN1*^*A431E*^, Supplementary Fig. 5A) than between mutations in different FAD genes but with the same iPSC reprogramming method (*APP*^*V717I*^* and PSEN1*^*A431E*^* or PSEN1*^*A79V*^* and PSEN1*^*N141I*^, Supplementary Fig. 5B-C).

Next, we sought to determine the disease endotypes associated with these three mutations. To this end, we first carried out the *fgsea* and CERNO enrichment tests with the GO Biological Process and Hallmark ontology databases to identify common and distinct disease endotypes. This revealed positive enrichment of gene sets related to cell cycle activation and dedifferentiation to non-ectoderm lineages (e.g., Epithelial-Mesenchymal Transition (EMT)) common across all mutations (Fig. 1D, Supplementary Fig. 1D). Interestingly, neuronal maturation and neuron function gene sets (e.g., synaptic signaling) were negatively enriched in *PSEN1*^*A79V*^ and *APP*^*V717I*^, these programs were modestly upregulated in *PSEN2*^*N141I*^. We next used curated gene lists for the key endotypes [15] observed here and performed tSNE clustering for pseudo-trajectory analysis of the three mutations studied here and two other mutations with an earlier AAO (*PSEN1*^*H163R*^, 42–47 years [108] and *PSEN1*^*A431E*^, 36–53 years [9]). This approach demonstrates that while *PSEN1*^*H163R*^ has the most severe dysregulation across all endotypes, *PSEN1*^*A79V*^ is similar to *PSEN1*^*A431E*^; in contrast, *APP*^*V717I*^ and *PSEN2*^*N141I*^ are comparatively less severe (Supplementary Fig. 6).

To characterize the transcriptional regulation of these disrupted gene programs, we used ISMARA [67] (Fig. 1E) and DoRothEA [68] (Fig. 1F) to predict TF activity. ISMARA identified several regulators that are common with significant differential activities across all mutations associated with key endotypes, including early neuron lineage (NFIB, PAX6, NEUROD1, MEIS2) [109–112], axonal growth and synaptogenesis (CREB1) [113, 114], mitochondrial energy and neuron function (NRF1) [115, 116], non-ectoderm lineage (TEAD1/3) [117, 118], pluripotency (GATA3 and TAF1), cell cycle (E2F1/7, FOXM1, ID4) [119], and inflammation (PRDM1) [120]. The neural differentiation repressor REST [121] was particularly activated in *PSEN1*^*A79V*^ compared to *APP*^*V717I*^ and *PSEN2*^*N141I*^. TF regulon analysis using DoRothEA revealed similar differential activity of TFs related to cell cycle (FOXM1, MYC, E2F1/4), neuron lineage and function (ASCL1, REST, EGR1) [122, 123], neuron mitochondrial energy production (NRF1), and non-ectoderm lineage (TEAD2, TCF3, TFAP2C) [124].

**Fig. 2:**
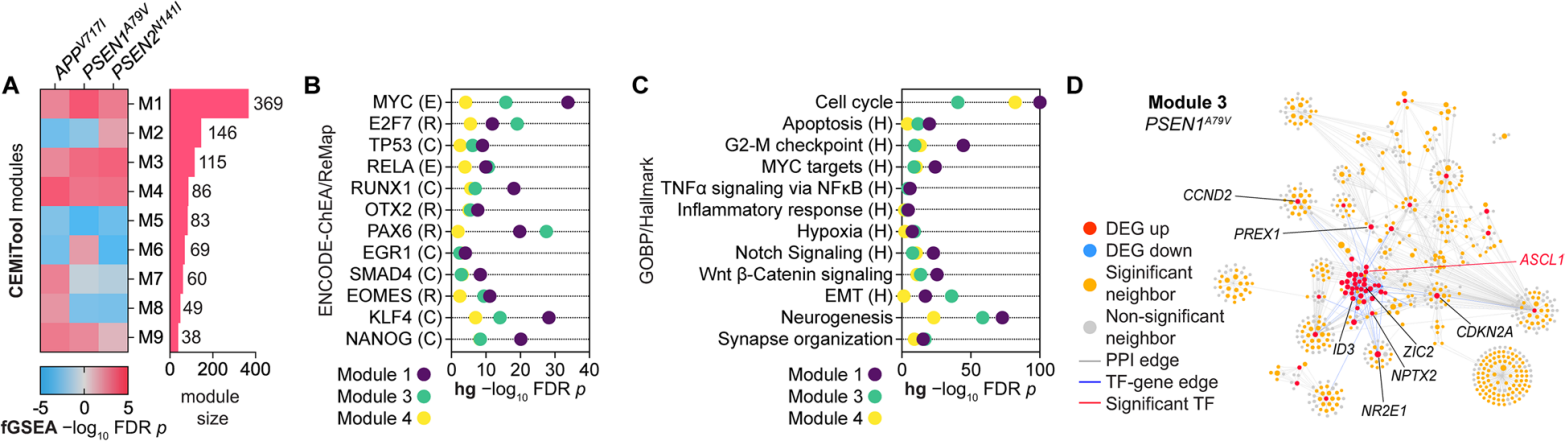
Co-expression module detection in FAD hiPSC-derived neurons*.* **A** Co-expression modules identified by CEMiTool module detection; left, *fgsea* enrichment of each module across the FAD mutations; right, gene size for each co-expressed module. **B**-**C** Hypergeometric enrichment of CEMiTool module and first neighbor genes using **B** ENCODE-ChEA Consensus and ReMap TF-gene target databases (ENCODE, E; ChEA, C; ReMap, R) or (**C**) GOBP and Hallmark ontology databases (Hallmark, H). **D** Combined PPI and TF-gene target networks of the CEMiTool co-expression module 3 for *PSEN1*^*A79V*^ hiPSC-derived neurons

Next, we performed co-expression module detection using the *CEMItool * [79] R package, followed by module enrichment in each mutation relative to NDC using *fgsea*. Nine functional co-expression modules were detected, with modules 1, 3 and 4 significantly enriched in all three mutations with a positive activity. In contrast, module 5 was enriched with a negative activity (Fig. 2A). Hypergeometric enrichment revealed an over-representation of genes associated with cell cycle, inflammation, non-ectoderm lineage, and early-stage neurogenesis (Fig. 2B-C). By integrating protein–protein interaction (PPI) and TF-target gene edges for module genes, neighboring genes, and key module TFs, key centroid genes for each module were identified, such as the lineage regulators *ASCL1* and *ZIC2* for module 3 (Fig. 2D, Supplementary Fig. 7).

**Fig. 3:**
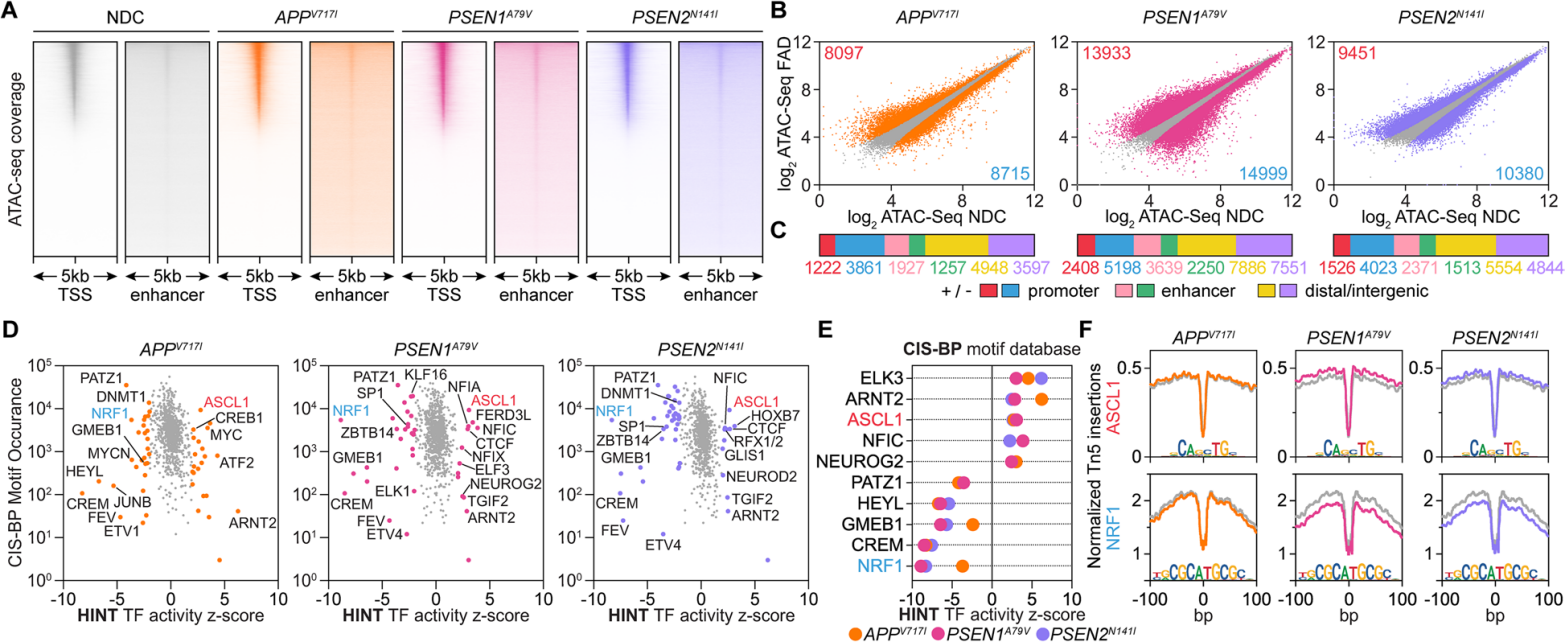
Regions of differential chromatin accessibility are enriched for transcriptional regulators and pathways mirroring gene expression signatures*.* **A** TSS and PEREGRINE enhancer heatmap coverage plots of Tn5-accessible chromatin in NDC, *APP*^*V717I*^, *PSEN1*^*A79V*^, and *PSEN2*^*N141I*^ hiPSC-derived neurons as determined by ATAC-seq. **B** Differential accessibility plots (log_2_FC) of ATAC-seq peaks for each FAD mutation relative to NDC (significant peaks: red, up; blue, down). **C** Annotation (promoter, PEREGRINE enhancer, or distal/intergenic) and directionality of significant differential ATAC-seq peaks for each FAD condition. **D** HINT TF footprinting analysis in all accessible ATAC-seq regions using the CIS-BP motif database to identify TFs with a change in footprinting activity. **E** Top differentially activated and repressed TFs across the FAD mutations based on HINT-ATAC footprinting analysis. **F** Tn5 insertion density in each FAD mutation relative to NDC around ASCL1 (top) or NRF1 (bottom) motifs as determined by HINT

To determine whether endotype transcriptional changes are driven by modulation of chromatin topology, we performed ATAC-seq (Fig. 3A, Supplementary Fig. 8) and assessed differentially accessible regions (DARs) within promoter or PEREGRINE [88] enhancer regions for each mutation relative to NDC (Fig. 3B-C). Next, we sought to identify TFs with differential activity associated with chromatin accessibility by motif footprinting using HINT-ATAC and motif enrichment with GimmeMotifs *maelstrom*. HINT analysis using the CIS-BP motif database identified decreased footprinting activity of TFs controlling neuron differentiation (HEYL, PATZ1) [125, 126], mitochondrial energy and neuron function (NRF1, GMEB1) [127], as well as synaptic plasticity (CREM) [128] and increased footprinting activity of early pro-neural TFs (ASCL1, NEUROG2, ARNT2) [129, 130] across all three mutations (Fig. 3D-F).

**Fig. 4:**
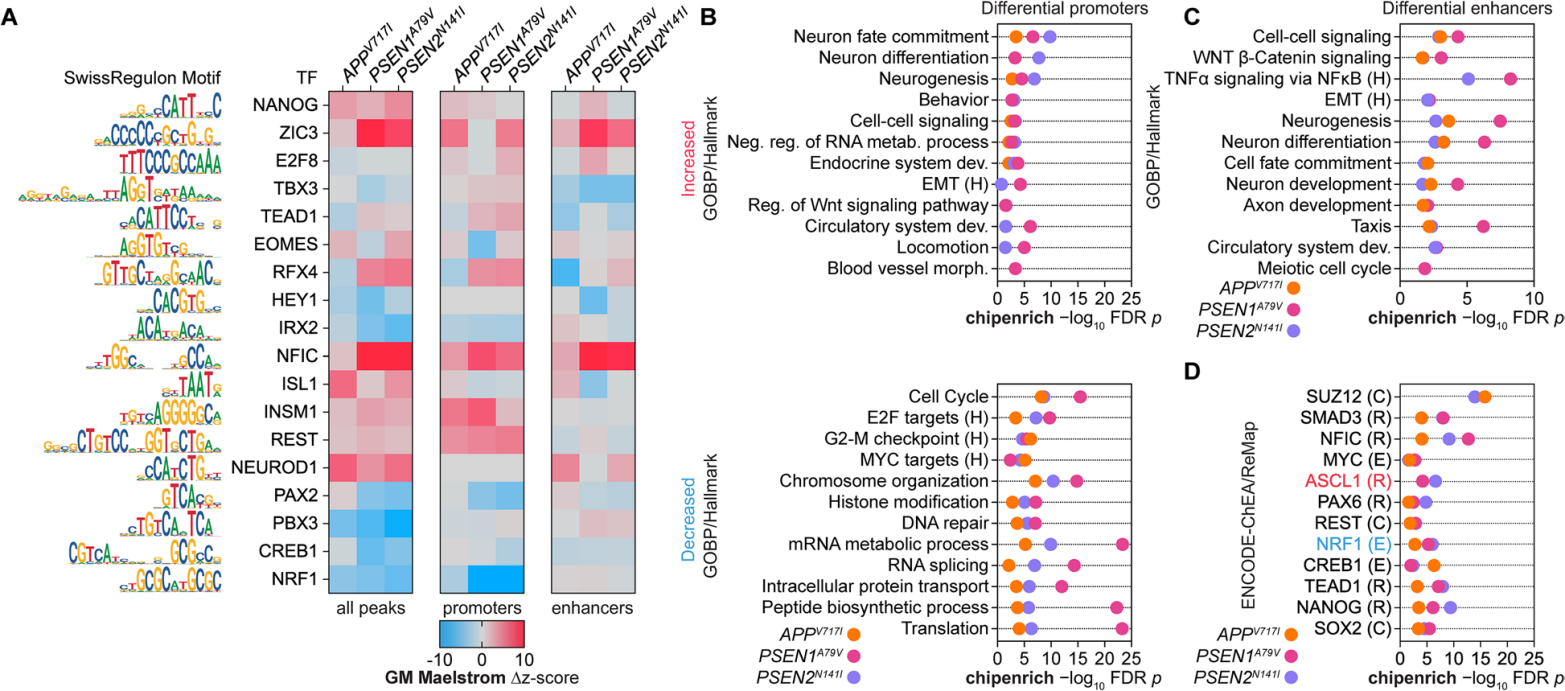
Transcription factor motif enrichment of chromatin accessibility reveals endotype-associated regulator differential activity. **A** TF motif enrichment of accessible ATAC-seq peaks (all peaks, promoter-associated peaks, and enhancer-associated peaks) using GimmeMotifs *maelstrom* with the SwissRegulon motif database. **B** *chipenrich* enrichment analysis of differentially accessible promoter-associated regions with increased accessibility (top) or decreased accessibility (bottom) using the GOBP and Hallmark ontology databases (Hallmark, H). **C**-**D** *chipenrich* enrichment analysis of differentially accessible enhancer-associated regions with differential accessibility using the (**C**) GOBP and Hallmark ontology databases or (**D**) ENCODE-ChEA Consensus and ReMap TF-gene target databases (ENCODE, E; ChEA, C; ReMap, R); FDR *p*-value < 0.05

For GimmeMotifs *maelstrom * [131] motif enrichment using the SwissRegulon motif database, we categorized consensus ATAC-seq peaks into three categories: all, promoter-associated, and enhancer-associated. This revealed increased accessibility at TF motif sites related to pluripotency (NANOG, ZIC3), cell cycle (E2F8), non-ectoderm lineage (TEAD1, TBX3, EOMES), early neuron lineage (NFIC, ISL1, INSM1, NEUROD1), and neuronal repression (REST). On the other hand, we observed decreased accessibility at TF motif sites related to late-stage neuron lineage (PAX2, PBX3) mitochondrial energy and neuronal function (NRF1), as well as axonal growth and synaptogenesis (CREB1) (Fig. 4A). To uncover the functional programs associated with chromatin accessibility change, we performed differential peak enrichment using *chipenrich* with GOBP and Hallmark pathway databases. Promoter DARs with increased accessibility were commonly enriched for early neuron lineage, non-ectoderm lineage dedifferentiation, and repression of RNA metabolism. Promoter DARs with decreased accessibility were commonly enriched for cell cycle, processes modifying the chromatin state, and proteasome-controlled processes (e.g., mRNA translation and metabolic process) (Fig. 4B). Enhancer DARs were enriched for similar processes, particularly for gene sets related to neuron differentiation, development, and non-ectoderm dedifferentiation (EMT, WNT β-Catenin Signaling) (Fig. 4C).

*chipenrich* with the ENCODE-ChEA and ReMap TF-gene target databases revealed enrichment for targets of TFs associated with pluripotency (NANOG, SOX2), early neuron lineage (NFIC, PAX6, ASCL1), neuronal repression (REST), axonal growth and synaptogenesis (CREB1), and neuronal mitochondrial function (NRF1) in FAD mutations (Fig. 4D). Finally, we examined whether FAD mutations caused differential accessibility near known AD risk variants (i.e., SNPs). To this end, we performed the intersection of published genetic variants in AD from multiple GWAS databases [98–100] with all DARs identified across the three FAD mutations. We identified 67 variants located within a DAR of at least one FAD mutation (Supplementary Fig. 9A) and 14 variants common across all three mutations (Supplementary Fig. 9B-C). We found the highest level of differential accessibility around AD genetic variants in *PSEN1*^*A79V*^ (*n* = 42), followed by *PSEN2*^*N141I*^ (*n* = 27) and *APP*^*V717I*^ (*n* = 26) (Supplementary Fig. 9E, 10A-E). Genetic variants commonly occurring across all three mutations within DARs with increased accessibility include *FERMT2*, which directly interacts with APP to modulate its metabolism [132] and *APH1B*, which is associated with elevated levels of Aß deposition [133] and assembly of the gamma-secretase complex [134] (Supplementary Fig. 10G-H). In contrast, we observed decreased accessibility around genetic variants for *ABT1,* which can modulate plaque-associated microglial activation [135] and *CWC25,* whose silencing affects Tau-induced toxicity [136]. (Supplementary Fig. 10I-J). These results suggest that variations in these AD-associated genes (via SNPs or differential chromatin accessibility) may interfere with overall neuron development and metabolic processing in FAD.

**Fig. 5:**
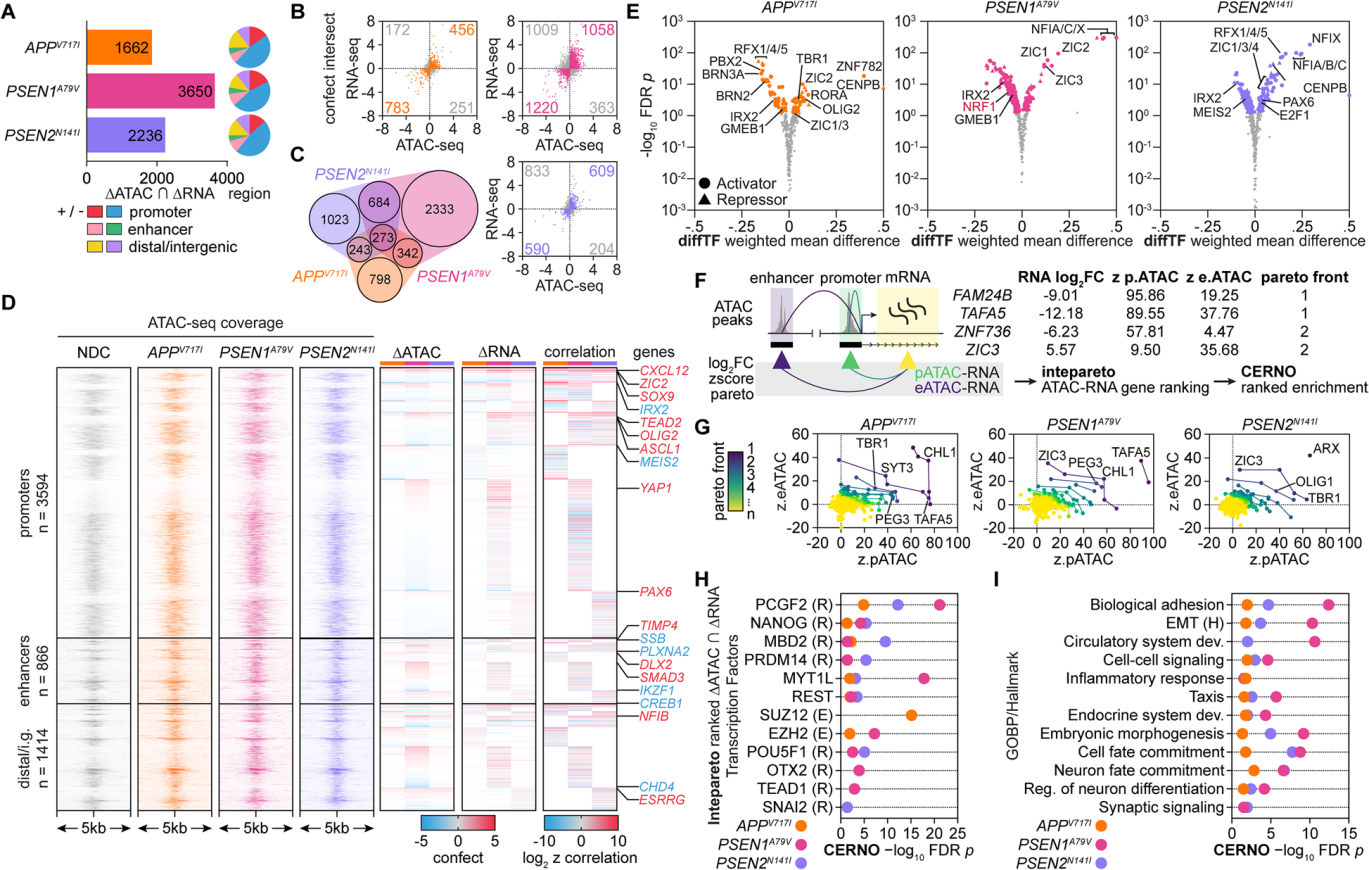
Chromatin accessibility change drives differential gene expression and dedifferentiation in FAD mutant hiPSC-derived neurons. **A** Differential ATAC-seq peaks with corresponding differential gene expression change in *APP*^*V717I*^, *PSEN1*^*A79V*^, and *PSEN2*.^*N141I*^ hiPSC-derived neurons relative to NDC; right, annotation (promoter, PEREGRINE enhancer, or distal/intergenic) and direction of differential ATAC-seq peak change. **B** CONfident efFECT size (confect) of differential chromatin accessibility (ATAC-seq) and gene expression (RNA-seq) for significant genes (by ATAC-seq) using *topconfects*. **C** quasi-proportional Venn diagram overlap of genes with significant differential accessibility and gene expression change between the FAD mutations. **D** Union of all genes with significant differential accessibility and gene expression in three FAD mutations; left, peak-centered ATAC-seq coverage in all four conditions; right, differential confect score for ATAC-seq and RNA-seq for each gene (relative to NDC), with corresponding z-score correlation; far right, genes with high correlation and increased (red) or decreased (blue) expression and accessibility change. **E** TFs with differential activity based on chromatin accessibility change (ATAC-seq) around TF motifs and target gene expression change (RNA-seq) across the three FAD mutations relative to NDC using DiffTF with the CIS-BP motif database. **F**-**G** Schematic for *intepareto* ranking of genes characterized by ATAC-seq and RNA-seq to identify functional programs with the highest correlation of chromatin accessibility change and gene expression change; z-scores of log_2_FC change of ATAC-seq peak accessibility change (promoter- or enhancer-located) and log_2_FC of RNA-seq gene expression for each gene across all FAD mutations relative to NDC, followed by pareto optimization ranking for each gene and subsequent CERNO ranked geneset enrichment test. **H**-**I** *intepareto*-CERNO ranked enrichment using the (**H**) ENCODE-ChEA Consensus and ReMap TF-gene target databases (ENCODE, E; ChEA, C; ReMap, R) and (**I**) GOBP and Hallmark databases (Hallmark, H)

**Fig. 6:**
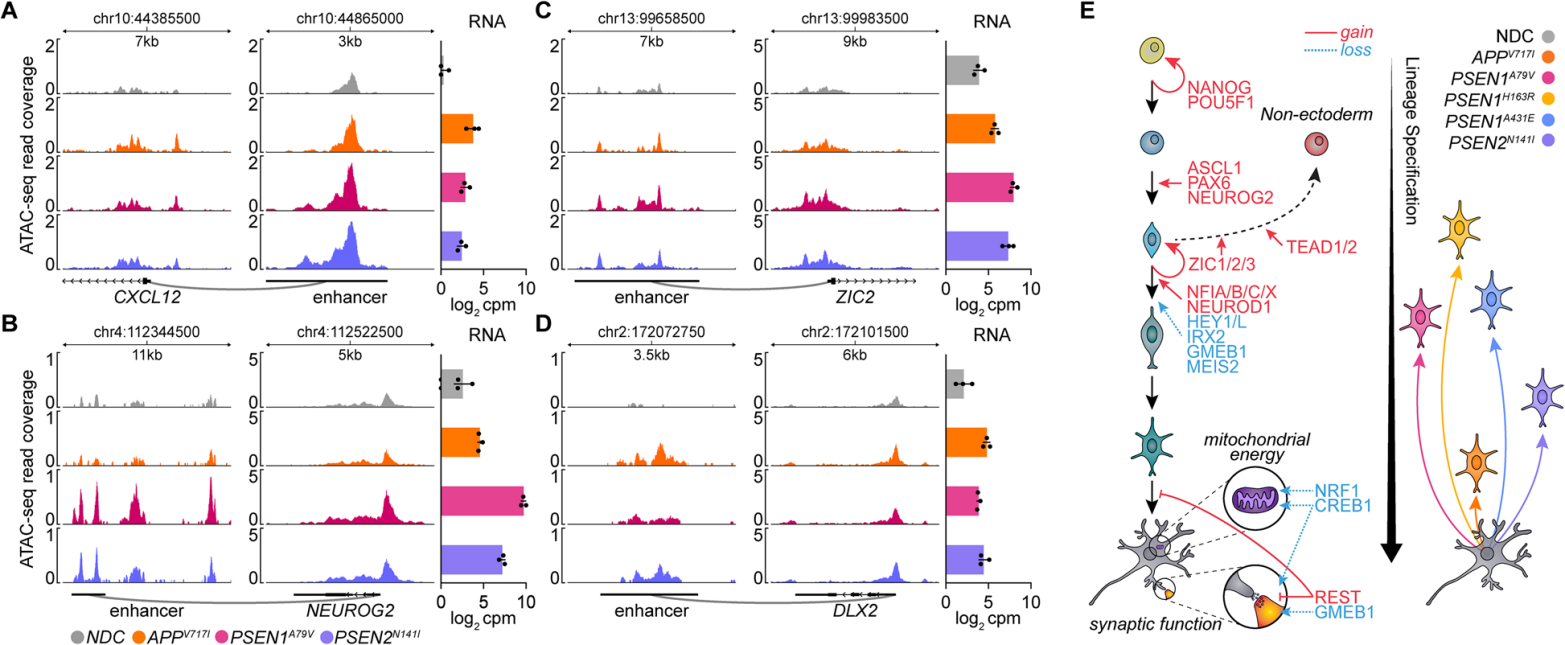
Endotype dysregulation driven by chromatin accessibility change or key regulator activity leads to precursor lineage state in FAD neurons. **A**-**D** ATAC-seq coverage plots (left) and RNA-seq expression (right) showing differential ATAC-seq peaks common across FAD mutant hiPSC-derived neurons occurring in promoter and enhancer regions for factors related to (**A**) inflammation (*CXCL12*), **B**-**C** neuronal development (*ZIC2*, *NEUROG2*), and (**D**) neuronal lineage (*DLX2*). **E** The hallmark disease mechanism in FAD mutations is dedifferentiation to a precursor-like state. Left, differentiation of pluripotent cell to a terminal neuron, with mechanistic TFs differentially regulated in FAD neurons (red, increased activity; blue, decreased activity). Right, qualitative comparison of severity of dedifferentiation across mutations in *PSEN1*, *PSEN2*, and *APP*

Previously, we demonstrated that chromatin accessibility changes precede and drive differential gene expression in *PSEN1* mutant neurons [15]; therefore, we sought to determine the correlation between differential accessibility and gene expression in the FAD neurons studied here. We found the intersect of genes with a non-zero CONfident efFECT size (confect) in both gene expression and chromatin accessibility using the *topconfects * [78] R package, revealing a substantial number of genes in each mutation, particularly in *PSEN1*^*A79V*^ (Fig. 5A-C). Most intersecting genes had DARs occurring in either promoter or enhancer regions, although some genes exhibited anti-correlated gene and accessibility change. To explore this further, we calculated the z-score correlation between gene expression and accessibility confect scores for all possible gene/peak pairs across the three mutations (Fig. 5D). This approach uncovered genes related to non-ectoderm dedifferentiation (*SOX9*, *TEAD2*, *YAP1*), early neuron lineage (*ZIC2*, *OLIG2*, *ASCL1*, *PAX6*), neuron differentiation (*IRX2*, *MEIS2*), and axonal growth and synaptogenesis (*CREB1*) with high correlation in at least one mutation. Next, we performed RNA-ATAC integration with diffTF to predict the differential activity of TFs using the CIS-BP motif database (Fig. 5E). By this approach, all three FAD mutations exhibited differential activity of factors involved in lineage development: increased activity of ZIC1/3 (activator) and decreased activity of IRX2 (repressor). Further, we observed activation of regulators involved in early neuron lineage (ZIC1/2/3 [137], NFIA/C/X [138], PAX6) but deactivation of those controlling late-stage neuron lineage (MEIS2) and mitochondrial energy and neuron function (NRF1, GMEB1) in both *PSEN1*^*A79V*^ and *PSEN2*^*N141I*^ mutations. Finally, we identified gene sets with a strong correlation between both promoter and enhancer accessibility change with differential gene expression using *intepareto * [103], followed by rank-based CERNO enrichment (Fig. 5F-G). This approach identified the targets of key TFs related to chromatin modification (PCGF2), pluripotency (NANOG), neuronal differentiation (MYT1L) (Fig. 5H) and ontological geneset processes related to lineage commitment, dedifferentiation (e.g., EMT), and neuronal differentiation commonly enriched in all three mutations with strongest correlation in *PSEN1*^*A79V*^ (Fig. 5I). In summary, this integration of RNA-seq and ATAC-seq demonstrates how the modulation of key disease endotypes, particularly reprogramming of non-ectoderm and neuronal lineages, are orchestrated via concerted chromatin and transcriptional changes. Indeed, the differential chromatin accessibility at both promoter and enhancer regions associated with transcriptional change for key endotype marker genes (*CXCL12, DLX2*; neuron function and lineage, respectively*)* and transcriptional regulators with predicted differential activity (*ZIC2*, *NEUROG2*; neuron lineage) highlight the correlation between chromatin accessibility, gene expression, and subsequent regulator activity change (Fig. 6A-D; Supplementary Fig. 9).

While many drugs developed to treat Alzheimer’s disease targeted Aβ peptide levels and amyloid plaques, the recent development of AD therapeutics has focused on modifying the disease at the pathway and cellular function level [104, 139]. Identifying commonly dysregulated endotypes across FAD mutations presented here allows us to explore the repository of existing or in-development drugs for potential endotype-targeting ability. To this end, we explored the Common Alzheimer’s Disease Research Ontology (CADRO)-based drugs and FDA-approved drugs. Here, we integrated FAD-associated enriched pathways and genes from our RNA-seq and ATAC-seq integrative analysis to find potential drug targets that could serve as therapeutic remedies for FAD. Using this approach, we identified CADRO-based drug agents based on a subset of commonly enriched FAD mutation pathways (*n* = 10) (Supplementary Fig. 11A) related to inflammation, synaptic plasticity, and neuroprotection in drug trial phases 1 and 2 (Supplementary Fig. 11B-C) and neurotransmitter receptors in drug trial phases 2 and 3 (Supplementary Fig. 11C-D). Predicted CADRO-based drug targets for such pathways are found most commonly in Phase 2 (*n* = 43; Figure S8C), with more overlapping relationships occurring among 36 drug target candidates related to synaptic plasticity and neuroprotection. This includes Fosgonimeton, an AD regenerative therapy drug that could potentially reverse synaptic disconnections and neuronal loss [140] (Supplementary Fig. 12D). Phase 1 (*n* = 16; Supplementary Fig. 12A) drugs with overlap of FAD endotypes include NNI-362, a therapy stimulator for p70S6 kinase phosphorylation that promotes neuron differentiation [141] and Allopregnanolone, a neurosteroid that promotes neurogenesis via GABA receptor complex activation on neural stem cells [142] (Supplementary Fig. 12B). A similar level of overlap was found with Phase 3 drugs (*n* = 15; Supplementary Fig. 12E), with neurotransmitter receptor candidates such as Donepezil, an acetylcholinesterase (AChE) inhibitor that helps remedy loss of functioning cholinergic neurons and improves cognitive decline in AD patients [143] (Supplementary Fig. 12F). When evaluating genes with both differential chromatin accessibility and gene expression (DAR/DEG), the highest number predicted drug target candidates in common FAD mutations occur in the promoter up category (*n* = 144; Supplementary Fig. 13A), followed by PEREGRINE-enhancer up (*n* = 76; Supplementary Fig. 14A), promoter down (*n* = 45; Supplementary Fig. 13C), and finally PEREGRINE-enhancer down (*n* = 6; Supplementary Fig. 14C). Potential drugs include Sunitinib, a tyrosine kinase inhibitor (similar to Donepezil) that remedies cognitive impairments [144], Bosutinib, a dual Abelson/Src inhibitor that promotes autophagy to remove Aβ protein aggregates [145], and Ruxolitinib, a JAK1/2 inhibitor to alleviate neuroinflammation and delay gliogenesis [146, 147] Supplementary Fig. 13B, 13D and 14B, 14D).

Our analysis of FAD mutations in *PSEN1*, *PSEN2*, and *APP* has focused exclusively on neurons, a key brain cell type affected in Alzheimer’s disease. However, recent research has highlighted the contributions to disease progression by alternative brain cell types, including microglia and astrocytes. We surmised that FAD astrocytes, a cell type that shares precursor lineage with neurons, may exhibit gene expression changes and associated endotypes similar to FAD neurons. To this end, we analyzed a previously published RNA-seq dataset on iPSC-derived astrocytes from patients harboring the *PSEN1ΔE9* FAD mutation [42]. Differential analysis of *PSEN1ΔE9* astrocytes relative to isogenically-corrected background astrocytes revealed 2513 upregulated and 2485 downregulated genes (Supplementary Fig. 15A-B). Interestingly, *fgsea* geneset enrichment using the GOBP and Hallmark databases showed positive enrichment of genesets related to cell cycle, inflammation, and chromatin remodeling, strikingly similar to the endotypes we observed in FAD neurons (Supplementary Fig. 15C-E). Furthermore, this revealed the downregulation of axonome assembly and cilium function, the loss of which is involved in mitochondrial [148] and cell–cell signaling dysfunction [149]. Surprisingly, the dedifferentiation process EMT was significantly downregulated in astrocytes, in contrast with the activation of EMT observed across FAD neurons.

## Discussion

Here, we present for the first time a direct comparison of representative mutations for each of the three genes associated with autosomal-dominant familial AD by profiling the respective transcriptomic and chromatin states in patient-derived iPSC neuron models. While most Alzheimer’s disease cases are sporadic, FAD mutations in *PSEN1*, *PSEN2*, and *APP* are nearly completely penetrant. Mutations in *PSEN1* are the most prevalent and tend to cause the earliest AAO (30 – 50 years), in contrast to the less common mutations in *APP* and *PSEN2*, which have a relatively later AAO. However, the specific mutations studied here, *PSEN1*^*A79V*^, *PSEN2*^*N141I*^, and *APP*^*V717I*^, each demonstrate an AAO around 55 years, yet the mechanistic avenues through which they arrive at the AD state have both subtle and substantial differences. By integrating gene expression measurements with chromatin accessibility, we identified the disease-associated cellular programs (i.e., endotypes) where gene dysregulation is driven by modulation of chromatin landscape changes and the key upstream regulators (i.e., TFs) that control them. Further, we describe the extent to which these mutations with late AAO exhibit common and distinct endotype dysregulation in terms of severity and direction, as observed in *PSEN1* mutations with an earlier AAO [15]. This integrative approach revealed common endotypes in all three mutations: dedifferentiation of a mature neuron to a less differentiated quasi-neuron state, inflammation, dysregulation of synaptic signaling, and repression of mitochondrial function and mRNA processing. The changes in these endotypes are due to the differential activity of common regulators; for example, in the case of non-ectoderm and early neuron lineage, the activation of ZIC [137, 150] family members and ASCL1 contribute to the genesis of these endotypes and the subsequent lineage state reversion. Concomitantly, we observed activation of the neural repressor REST, resulting in the downregulation of genes involved in synaptic maturation and function; deactivation of the transcriptional activator NRF1, which has been well-described as a key regulator of genes associated with mitochondrial energy function, metabolism, synaptic transmission and cell cycle regulation [116, 151, 152]. Importantly, the loss of NRF1 activity leads to mitochondrial dysfunction, decreased synaptic function, and neurodegeneration, as previously reported in AD [151, 153]. These REST- and NRF1-controlled target genes were found to have decreased chromatin accessibility in our ATAC-seq analysis (Fig. 6E). This combination of mitochondrial dysfunction and repression of synaptic maturation and function can ultimately drive degeneration, leading to synaptic loss. The relevance of this loss of neuron lineage state and synaptic function orchestrated through chromatin remodeling is readily apparent: the hallmark clinical manifestation of Alzheimer’s disease, both in familial and sporadic forms, is cognitive decline; underlying these outward symptoms are the strongly correlated pathobiological features of synaptic dysfunction and loss. Furthermore, the concept of dedifferentiation in AD neurodegeneration is not new; it has been posited that dysregulation of synaptic plasticity is inextricably connected to the loss of neuronal lineage state, re-entry into the cell cycle, and reversion to a precursor-like state in AD [154, 155]. Others have previously demonstrated these mechanisms in neuron models of sporadic AD [16], while we have demonstrated them in neuron models of *PSEN1* AD [15]; here, we present evidence that this is a feature of familial AD caused by mutations in *APP* and *PSEN2* as well*.*

Amongst the common disease endotypes, the severity of dysregulation of each disease endotype differed amongst the three mutations, as evidenced by the statistical significance of both functional enrichment of geneset terms and the activity change of key TFs regulating them. Not surprisingly, we identified the same disease endotypes in the *PSEN1*^*A79V*^ mutation as we previously uncovered in a larger study on *PSEN1* mutations [15]. However, the magnitude of dysregulation was dampened in *PSEN1*^*A79V*^ neurons (Figure S2A-E), perhaps explaining its later AAO relative to other *PSEN1* mutations with an earlier AAO, such as *PSEN1*^*H163R*^ and *PSEN1*^*A431E*^*,* which demonstrate higher magnitudes of dysregulation. Amongst the two less common FAD mutation types, *APP*^*V717I*^ aligns with the directional change in key endotypes of the *PSEN1* mutations, albeit with a dampened magnitude and significance. In contrast, *PSEN2*^*N141I*^ demonstrates a similar magnitude of endotype dysregulation as *PSEN1* mutations, albeit the direction of change for later stages of neuron lineage and function is opposite to the *PSEN1* and *APP* mutations (Supplementary Fig. 2). The consequence of the increase in neuron lineage and function observed in the *PSEN2* mutation could be a related to hyperexcitability or accelerated maturation, ultimately resulting in a non-terminal neuronal state. Further, the top regulator for some endotypes differed between mutations. For example, we observed that OLIG2 is particularly activated in *APP*^*V717I*^, whereas differential activation of PAX6 is more specific to *PSEN2*^*N141I*^*,* demonstrating a common activation of early neuronal lineage via distinct regulators. This type of endotype heterogeneity is not uncommon in AD, as heterogeneity is observed to a far greater extent in SAD [156].

The dominant theory of AD genesis has been that aberrant Aβ processing leads to a cascade of plaques, tangles, and subsequent onset of cognitive decline. Although the protein product of the FAD genes is either the catalytic component (PSEN1, PSEN2) or the substrate (APP) in the proteolytic processing of APP into amyloid peptides, whether the accelerated development of plaques and tangles are indeed the cause of early onset AD or rather a consequence of disease has remained unclear. Further complicating the matter is that although *PSEN1* and *PSEN2* are homologs and carry out somewhat analogous functions within the gamma-secretase complex, recent evidence suggests that there are distinct pools of *PSEN 1*- and *PSEN2*-containing gamma-secretase complex, both in terms of the cellular compartment location as well as holoenzyme membership: *PSEN1* is expressed ubiquitously and localizes broadly to gamma-secretase complexes throughout the cell while *PSEN2-*containing gamma-secretase complex is localized primarily to late endosomal/lysosomal compartments [157]. This may account for the greater similarity observed between the *PSEN1*^*A79V*^ and *APP*^*V717I*^ mutations compared to the *PSEN1*^*A79V*^ and *PSEN2*^*N141I*^ mutations with respect to the direction of dysregulation for neuronal lineage and function. In contrast, the level of dysregulation observed in the cell cycle and inflammation is similar between the *PSEN1*^*A79V*^ and *PSEN2*^*N141I*^ mutations*.* There are at least two possible reasons for the differential effect of *PSEN1* and *PSEN2* mutations within disease endotypes: first, while Aβ processing is aberrant in both the *PSEN1* and *PSEN2* mutations, the proximity of presenilin-containing gamma-secretase complexes to APP pools likely plays a role in the levels of Aβ peptide species as well as the processing of alternative substrates; and second, while neurons are particularly affected by *PSEN1* mutations, *PSEN2* may be the preferential gamma secretase catalytic component in microglia [21, 158, 159]. These reports suggest that the inflammatory endotype modestly enriched in the *PSEN2*^*N141I*^ neurons may be more apparent in the canonically inflammatory microglial cell type. Interestingly, our reanalysis of a previous study revealed that this activation of inflammatory genes was also observed in patient-derived astrocytes with the *PSEN1ΔE9* FAD mutation. This was accompanied by an activation of genes associated with cell cycle and chromatin remodeling and repression of genes associated with cell–cell signaling, suggesting that the FAD disease endotypes identified in our iPSC-derived neuron model system are also relevant for alternative cell types. Others have recently demonstrated that the Aβ profile ratio (i.e., (Aβ37 + Aβ38 + Aβ40)/(Aβ42 + Aβ43)), a measure of the processivity of the gamma-secretase enzyme, correlates with AAO; by this measure, *PSEN1*^*A79V*^ has a modestly reduced Aβ profile ratio which leads to a late AAO relative to other *PSEN1* mutations [9]. In vitro measurements of gamma-secretase processivity demonstrated that *PSEN1* and *PSEN2* mutations modulate the ε-endoproteolytic cleavage step but not consistently in the same direction across mutations, whereas the carboxypeptidase-like γ-cleavage step was consistently altered towards premature release of longer Aβ peptide forms (i.e., Aβ42 and Aβ43). Mutations in *PSEN1* and *PSEN2* result in varying degrees of alterations in either the start site or the exit site of cleavage, or both, resulting in differential alteration of Aβ ratios. In contrast, *APP* mutations affect the docking position at the ε-cleavage site but not the γ-cleavage [160, 161]. Although mutations in *PSEN2* cause a similar decrease in the Aβ profile ratio, the limited distribution of *PSEN2* could cause the later AAO observed. Furthermore, while the two presenilins share 67% sequence homology and PSEN1 may play a functional compensatory role in *PSEN2* mutations, the inverse does not appear to be true in *PSEN1* mutations [21, 162]. For example, the *PSEN1*^*N135D*^ has a documented mean AAO of 34 years, while its sister variant in *PSEN2, PSEN2*^*N141D*^ (similar to the *PSEN1*^*N141I*^ studied here), has an AAO of 59 years [9].

The differences in cellular location and cell type are not the only possible explanations of differential endotype dysregulation between the mutations in each of the three FAD genes; there are over 150 alternative substrates of gamma-secretase that have been identified in addition to APP and altered processivity of the gamma-secretase enzyme may have myriad effects via these substrates [13]. Some key examples are Notch, a key regulator of Wnt signaling that promotes proliferation during neurogenesis but is also repressed during neural differentiation [13], and p75NTR, a neurotrophin receptor and cell cycle regulator which has been proposed to be involved in AD via multiple modes of action including neuronal growth regulation, differentiation and cellular survival, and cellular senescence [163, 164]. Structural changes due to mutations in *PSEN1* (and, to a lesser extent, its cellular localization, *PSEN2*) could also play a role here; endoproteolytic processing at the ε-cleavage site of alternative substrates like Notch are also variably affected depending on mutation type [160]. This is reflected in our co-expressed module analysis: module 1 is particularly enriched for Notch and WNT signaling pathways and transcriptionally controlled by the pro-EMT (dedifferentiation) regulator RUNX1 [165]. While module 1 is positively enriched in all three mutations, the strongest enrichment is observed in *PSEN1*^*A79V*^. Therefore, mutations in *PSEN1* may exhibit stronger dysregulation in certain disease endotypes relative to those in *PSEN2* or *APP* due to the mutation-induced effects on substrates alternative to APP.

The ultimate goal of characterizing the regulatory transcriptome of FAD is to identify potential disease endotypes and their regulators that may be optimal for disease modification by drug therapeutics. We found that the DEG/DAR-based predicted drug candidates and the CADRO-based clinical drug therapies associated with common enriched FAD pathways address the AD endotypes we present here. This approach highlights therapeutic remedies for such endotypes, paving the way for advancing drug therapeutics for FAD.

A significant challenge to studying the gene regulatory mechanisms associated with the onset and development of AD is the suitability of model systems that faithfully recapitulate disease-associated dysregulation in the patient’s brain. We previously reanalyzed one of the few publicly available transcriptomic studies on FAD mutations in *PSEN1*, observing repression of neuronal lineage and synaptic function concomitant with activation of cell cycle and dedifferentiation programs [15, 166]. The complete penetrance of familial AD mutations and the advent of patient-derived neurons offer the ability to capture disease mechanisms observed in post-mortem brains [15, 16] in a non-invasive manner. However, there is a relative immaturity of patient-derived neuron cultures compared with the developed brain, such that all disease-associated neuron subtypes are not likely fully represented in the model system we have described here. While the advent of new initiatives has increased the availability of engineered iPSC lines for mutations spanning many types of dementia [167], there is still a limited amount of mutations available, as well as patient-derived lines from different patients with the same mutation. Furthermore, while a considerable amount of RNA-seq data is available for large cohorts of SAD patients, this is not the case for FAD to corroborate iPSC-derived neuron profiling for all potential mutation types. Recent work has highlighted the role of non-neuronal cell types in the onset and progression of AD, so future studies must focus on the interplay across brain cell types via patient-derived organoid systems [168] to model FAD.

The common hallmark phenotype in familial and sporadic AD is progressive cognitive decline. The combination of the underlying endotypes described here leads to a defective neuron network and diminished cognition. The observed repression of neuron differentiation and function concomitant with the activation of early neuron lineage in *APP*^*V717I*^ and *PSEN1*^*A79V*^ via the differential activity of neuronal precursors (e.g., PAX6 and ASCL1 activation) and neuronal and mitochondrial repression (e.g., activation of REST, deactivation of NRF1), respectively, are indicative of reversion to a less defined, yet early neuronal state (Fig. 6E). This, combined with activation of non-ectoderm lineage dedifferentiation and cell cycle dysregulation, indicates a reversion to a precursor-like state. While dedifferentiation is a shared feature between the *PSEN1*, *PSEN2*, and *APP* mutations, the differential endotype manifestation arising from the combination of shared and unique regulatory mechanisms poises each mutation type to a distinct precursor state along a neuronal lineage landscape. Ultimately, the common and unique endotypes identified here and the key regulators driving their differential activity can serve as a basis for understating the molecular mechanisms of AD that may aid in therapeutic development.

## Supplementary Information

Supplementary Material 1.

Supplementary Material 2.

Supplementary Material 3.

## Data Availability

RNA-seq and ATAC-seq data are available at the NCBI GEO under the SuperSeries accession GSE206603. Normalized count matrices for RNA-seq and ATAC-seq data are provided in Supplementary files 2 and 3. Cell lines are available upon request from Dr. Celeste Karch at Washington University in St. Louis (karchc@wustl.edu) and Dr. Shauna Yuan at University of Minnesota (syuan@umn.edu). Code for all analysis is available for download from https://github.com/SubramaniamLab/FAD-Multiomics-Manuscript and 10.5281/zenodo.8267332.
