# Supplementary material for: Integrative multiomics reveals common endotypes across PSEN1, PSEN2, and APP mutations in familial Alzheimer’s disease
Source: Alzheimers Res Ther. 2025 Jan 4;17:5. doi: 10.1186/s13195-024-01659-6 (PMC11699654; doi:10.1186/s13195-024-01659-6)
Supplement: Supplementary file 1 — Supplementary Material 1. [file 13195_2024_1659_MOESM1_ESM.pdf]

Valdes *et al.* *Alzheimer's Research & Therapy* 2025
https://doi.org/10.1186/s13195-024-01659-6

Supplementary Materials for

Integrative multiomics reveals common endotypes across *PSEN1*, *PSEN2*, and *APP* mutations in familial Alzheimer's disease.

Valdes and Caldwell *et al.*

Corresponding author email: shankar@ucsd.edu

This PDF file includes:

Figs. S1 to S15

https://doi.org/10.1186/s13195-024-01659-6

A

iPSC-derived neurons

Cell Line	APOE Status	Age at Biopsy	Age at Onset	Sex	Mental Status at Biopsy	RNA RIN score	First reported in
NDC	2 / 3	85	-	M	Normal	7.1 - 7.3	Israel *et al.* 2012
APP^{V717I}	3 / 3	37	NA	F	Normal	3.7 - 6.1	this paper
PSEN1^{A79V}	3 / 4	-	NA	M	Normal	7.9 - 8.4	Karch *et al.* 2018
PSEN2^{N141I}	3 / 3	-	NA	F	Normal	7.8 - 8.2	Karch *et al.* 2018

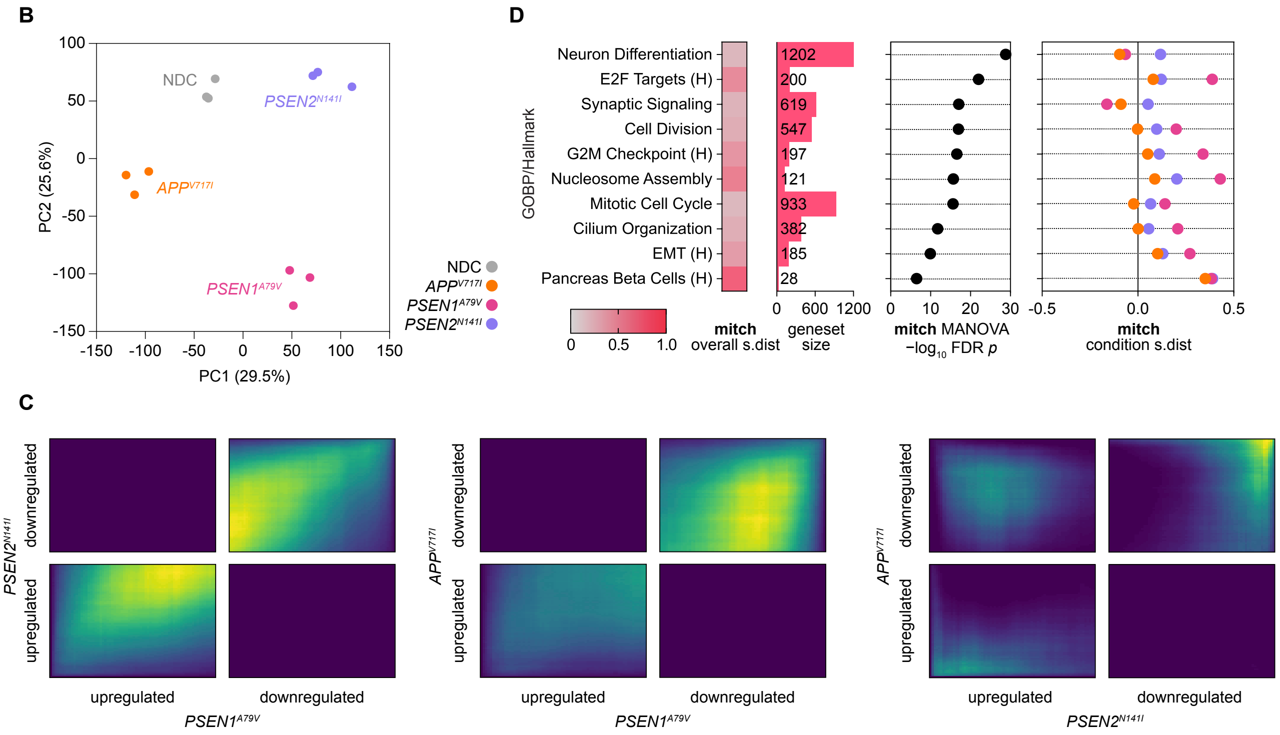

Supplementary Figure 1 *Characterization of NDC and FAD hiPSC-derived neurons*
A Non-demented control (NDC), *APP*^{V717I}, *PSEN1*^{A79V}, and *PSEN2*^{N141I} hiPSC lines differentiated into neurons in this study for RNA-seq and ATAC-seq. **B** Principal component analysis (PCA) of all sample groups using normalized RNA-seq data. **C** Rank Rank Hypergeometric Overlap (RRHO) analysis between the three mutation pairs using the *RRHO2* R package. **D** rank-MANOVA enrichment of Hallmark and GOBP genesets across FAD neurons using the *mitch* R package; left, overall enrichment (s. dist) of each geneset across all three mutations and geneset size; center, FDR *p*-value (*p*) of rank-MANOVA test across all three mutations; right, mutation specific enrichment direction and magnitude (s. dist).

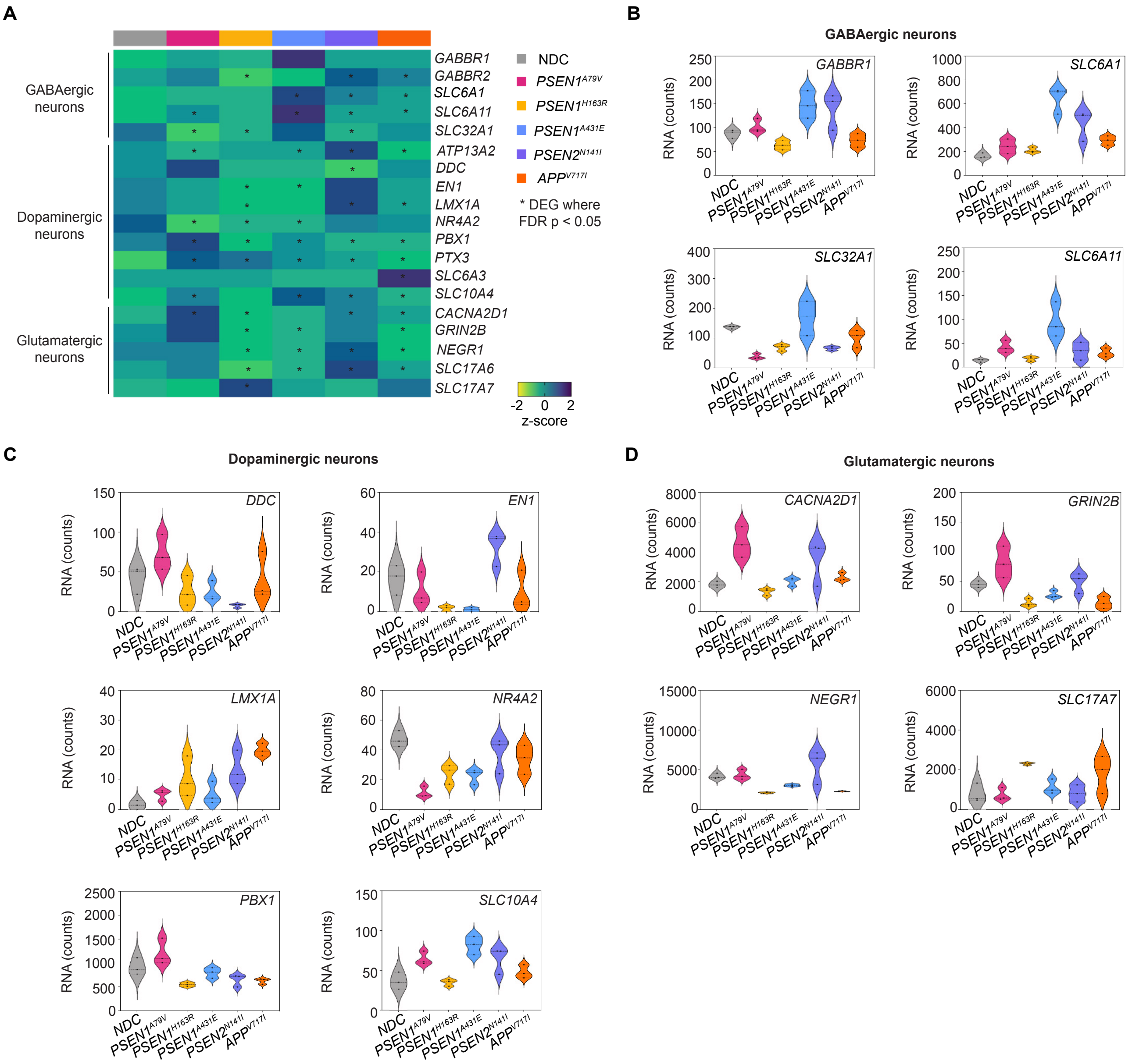

Supplementary Fig. 2 *Relative RNA-seq levels across NDC and FAD mutations show similar expression of marker genes for GABAergic, dopaminergic and glutamatergic neuron maturation*
A Hierarchical clustering heatmap of GABAergic, Dopaminergic, and Glutamatergic neuron markers using normalized and filtered expression data. Differentially expressed genes are indicated by * (FDR p-value < 0.05). Violin plots of gene expression levels across NDC and FAD mutations for **B** GABAergic neuron markers, **C** Dopaminergic neuron markers, and **D** Glutamatergic neuron markers.

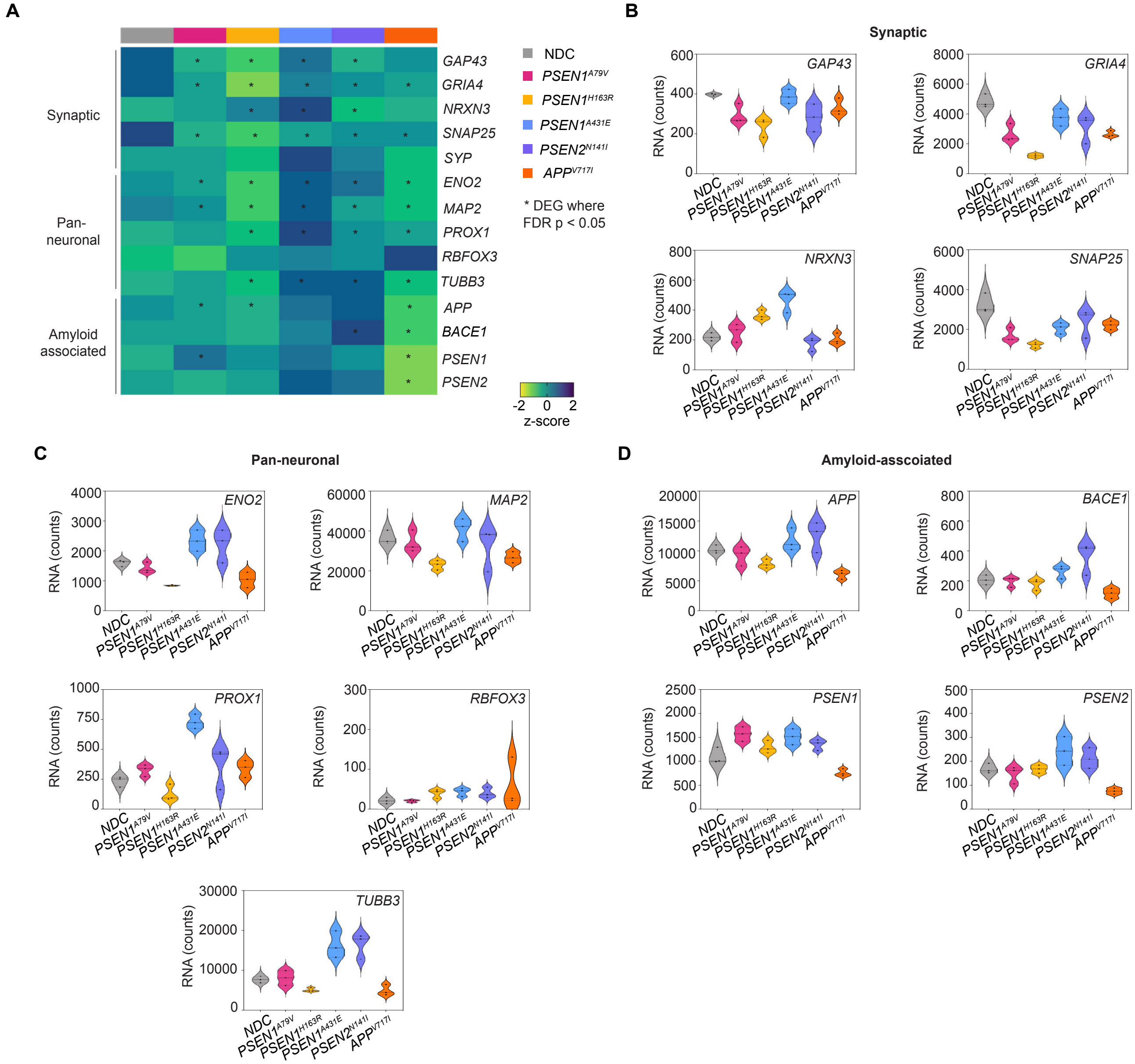

Supplementary Fig. 3 *Relative RNA-seq levels across NDC and FAD mutations show similar expression of synaptic, pan-neuronal, and amyloid-associated marker genes across NDC and FAD mutations*

A Hierarchical clustering heatmap of synaptic, pan-neuronal and amyloid-associated marker genes using normalized and filtered expression data. Differentially expressed genes are indicated by * (FDR p-value < 0.05). Violin plots of gene expression levels across NDC and FAD mutations for **B** synaptic neuron, **C** pan-neuronal, and **D** amyloid-associated markers.

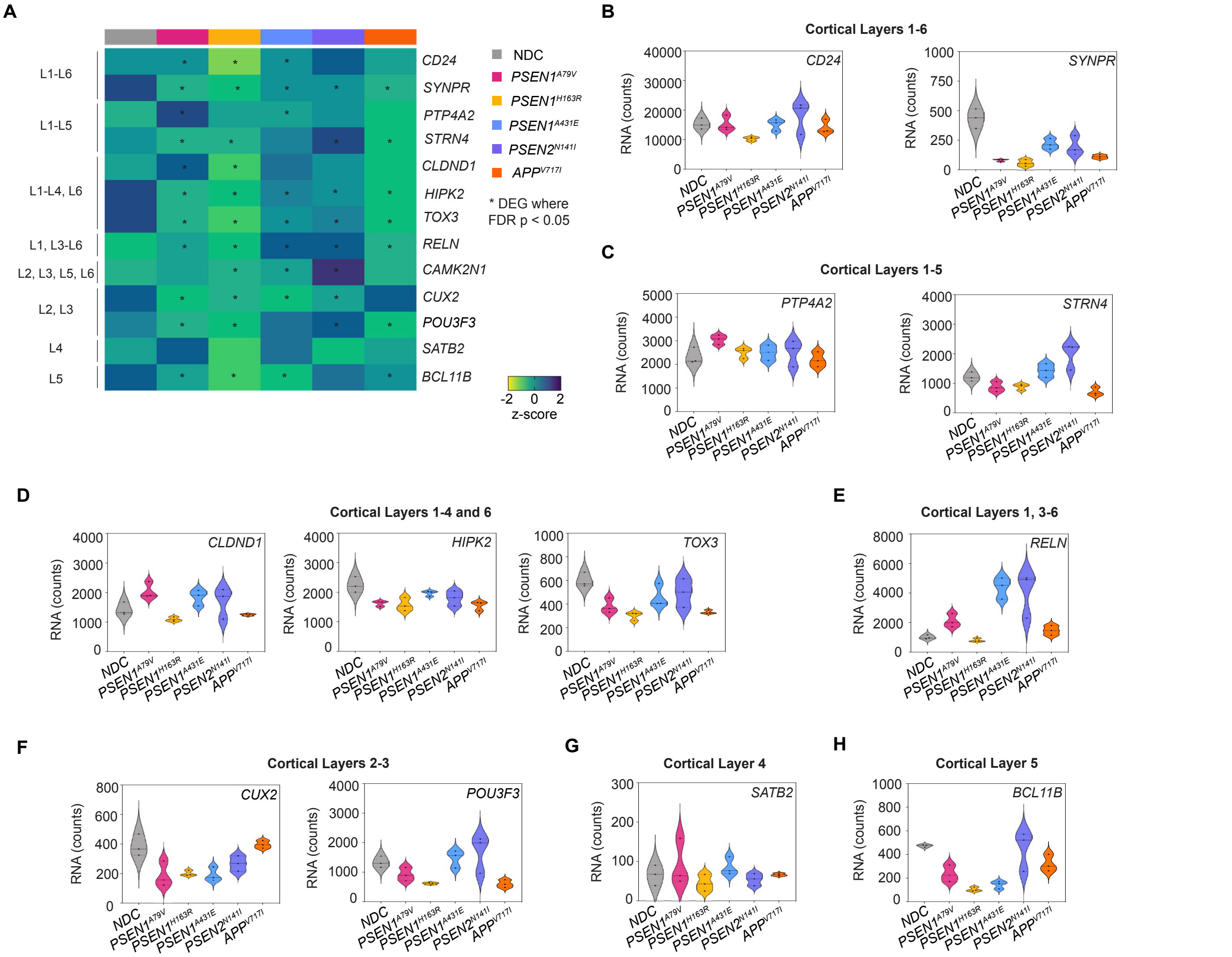

Supplementary Fig. 4 *Relative RNA-seq levels across NDC and FAD mutations show similar expression of marker genes associated with six different cortical layers*

A Hierarchical clustering heatmap of different neuron markers found in one or more cortical layers localized in layers 1-6 (i.e. L1-L6) using normalized and filtered expression data. Differentially expressed genes are indicated by * (FDR p-value < 0.05). Violin plots of gene expression distribution across NDC and FAD mutations for **B** neuron markers (*CD24*, *SYNPR*) found in all six cortical layers (i.e. L1-L6), **C** neuron markers (*PTP4A2*, *STRN4*) found in five cortical layers (i.e. L1-L5), **D** neuron markers (*CLDND1*, *HIPK2*, and *TOX3*) found in five cortical layers (i.e. L1-L4, L6), **E** a neuron marker (*RELN*) found in five cortical layers (i.e. L1, L3-L6), **F** neuron markers (*CUX2, POU3F3*) found in two cortical layers (i.e. L2-L3), **G** a neuron marker (*SATB2*) found in one cortical layer (i.e. L4), and **H** a neuron marker (*BCL11B*) found in one cortical layer (i.e. L5).

https://doi.org/10.1186/s13195-024-01659-6

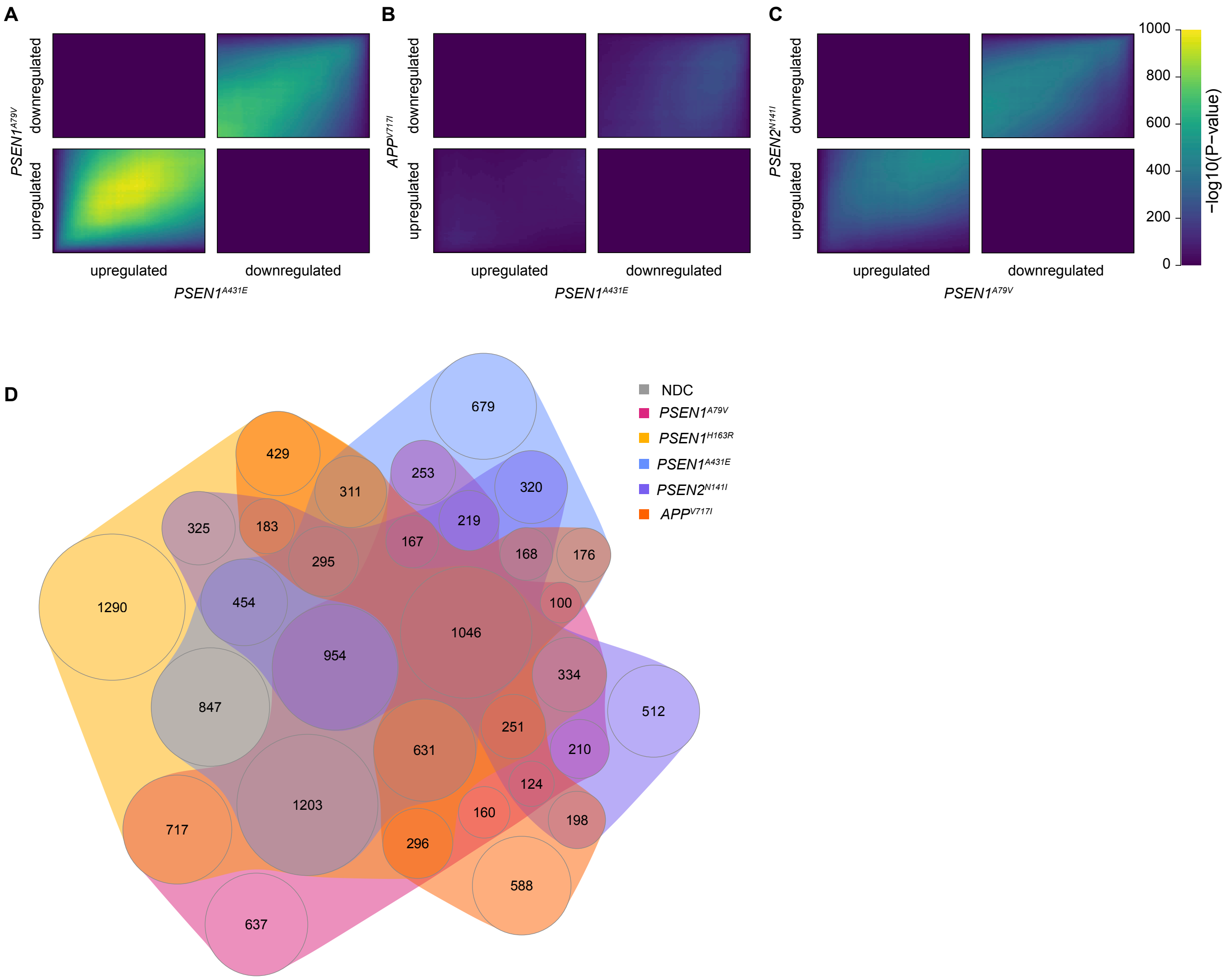

Supplementary Fig. 5 *RRHO analysis of FAD mutations demonstrates greater expression similarity between FAD mutation types compared with the reprogramming method*

Rank-Rank Hypergeometric Overlap (RRHO) analysis of **A.** two *PSEN1* mutations with different reprogramming methods: *PSEN1*^{A79V} (retroviral transduction) and *PSEN1*^{A431E} (episcopal), and two FAD mutations from different genes using the same reprogramming method: **B.** *APP*^{V717I} and *PSEN1*^{A431E} (episcopal) and **C.** *PSEN1*^{A79V} and *PSEN1*^{N141I} (retroviral transduction). **D.** nVenn diagram of DEG overlap across all 5 FAD mutations.

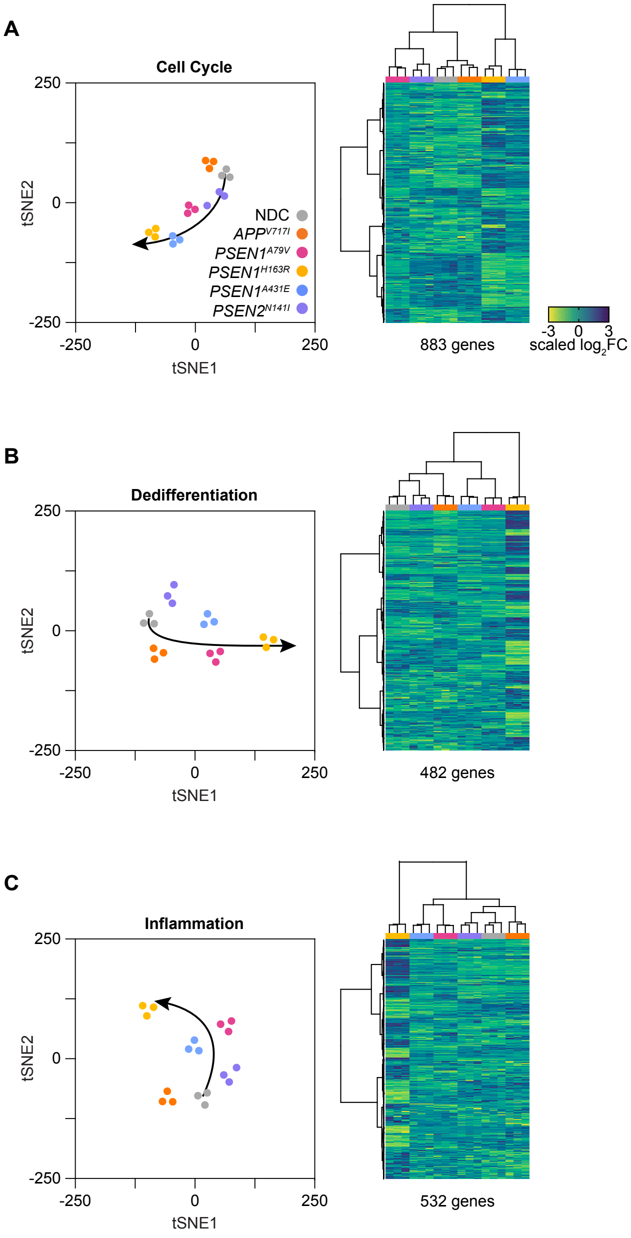

Supplementary Figure 6 *Pseudo-trajectories of FAD neurons for disease endotypes by* tSNE
A-E t-distributed stochastic neighbor embedding (tSNE) analysis of NDC and FAD neuron expression for genes involved in key disease endotypes (cell cycle, dedifferentiation, inflammation, neuron lineage, and neuron function); right, log_2FC RNA expression heatmap of corresponding endotype genes clustered by genotype expression profile.

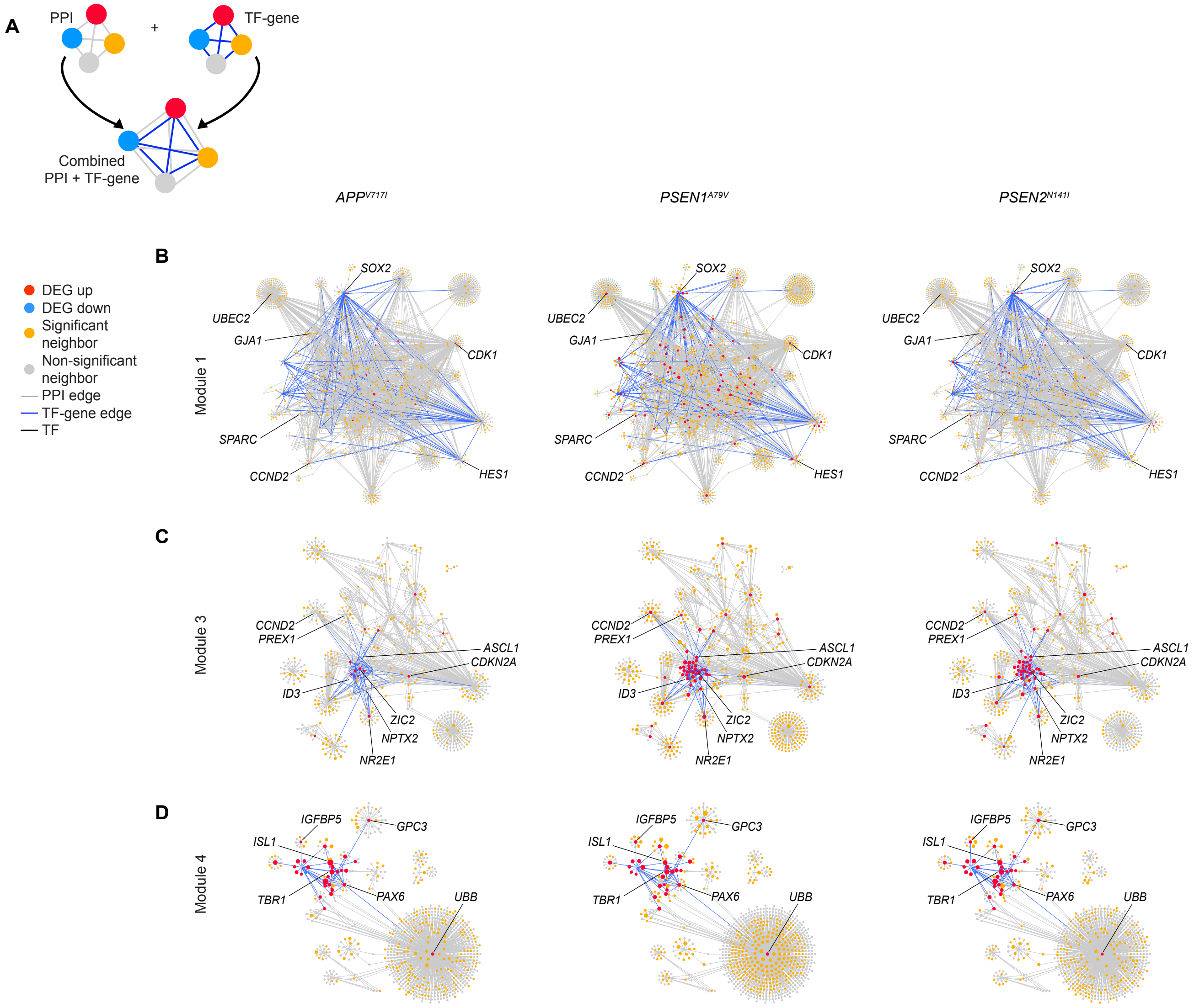

Supplementary Figure 7 *Regulatory interaction networks for key coexpression gene modules*
A Schematic demonstrating the embedding of protein-protein interaction and TF-gene networks together to create regulatory interaction networks for coexpression modules (red = DEG up; blue = DEG down; yellow = DEG neighbor gene; and grey = non-significant gene). **B-D** Regulatory interaction networks of CEMiTool coexpression modules. **B** M1; **C** M3; and **D** M4 for FAD mutant neurons (grey edges = PPI interactions; blue edges = TF-gene interactions).

https://doi.org/10.1186/s13195-024-01659-6

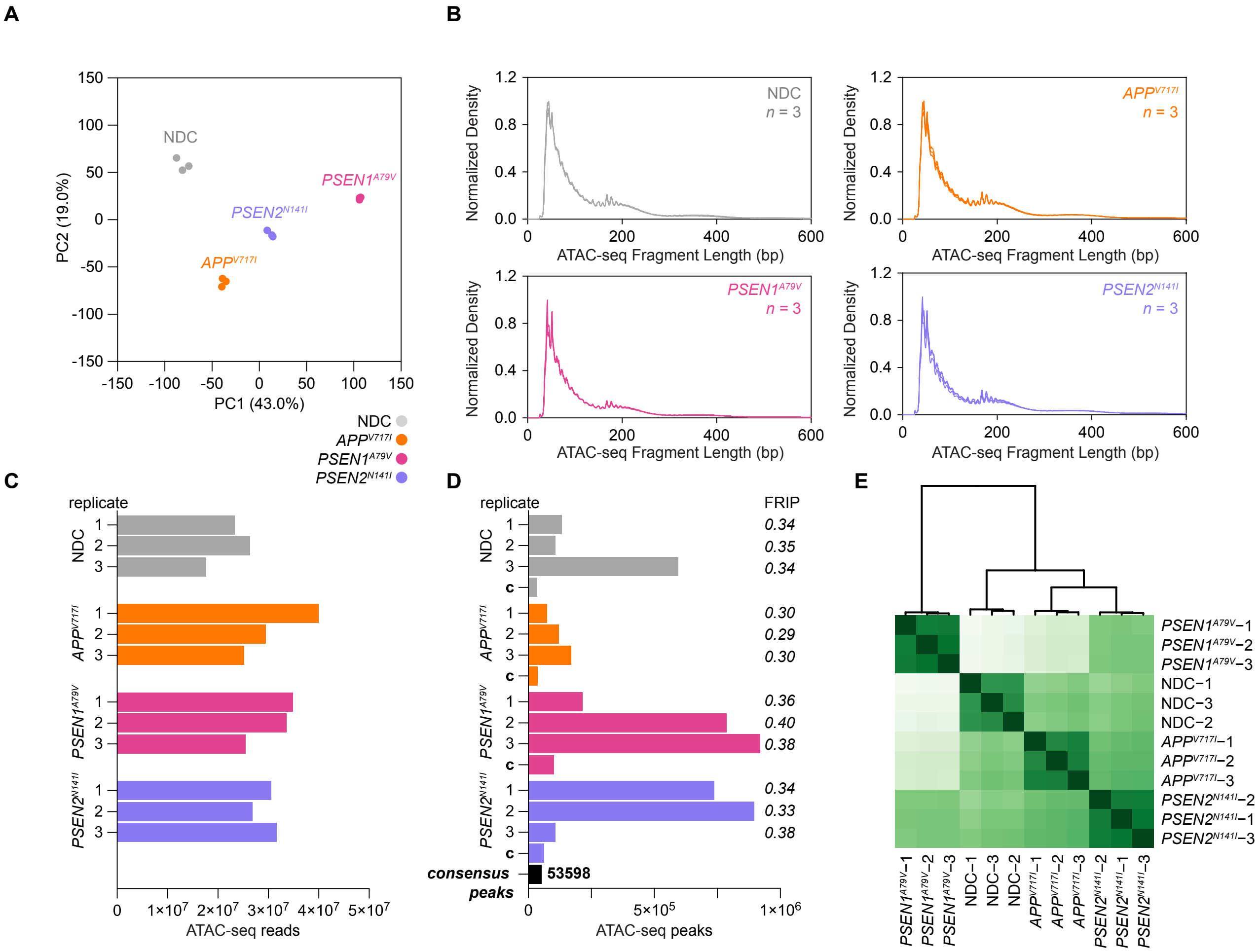

Supplementary Figure 8 *ATAC-seq processing of hiPSC-derived FAD neurons*
A Principal component analysis (PCA) of all conditions using normalized ATAC-seq data. **B** Insert size distribution of ATAC-seq insert sizes in NDC, *APP*^{V717I}, *PSEN1*^{A79V}, and *PSEN2*^{N141I} hiPSC-derived neurons. **C** Sample-specific read depth of ATAC-sequencing. **D** Individual replicate, condition consensus, and overall consensus peak list from ATAC-seq analysis; right, fraction of reads in peaks (FRIP) for each sample. **E** Correlation of peak density across all ATAC-seq samples.

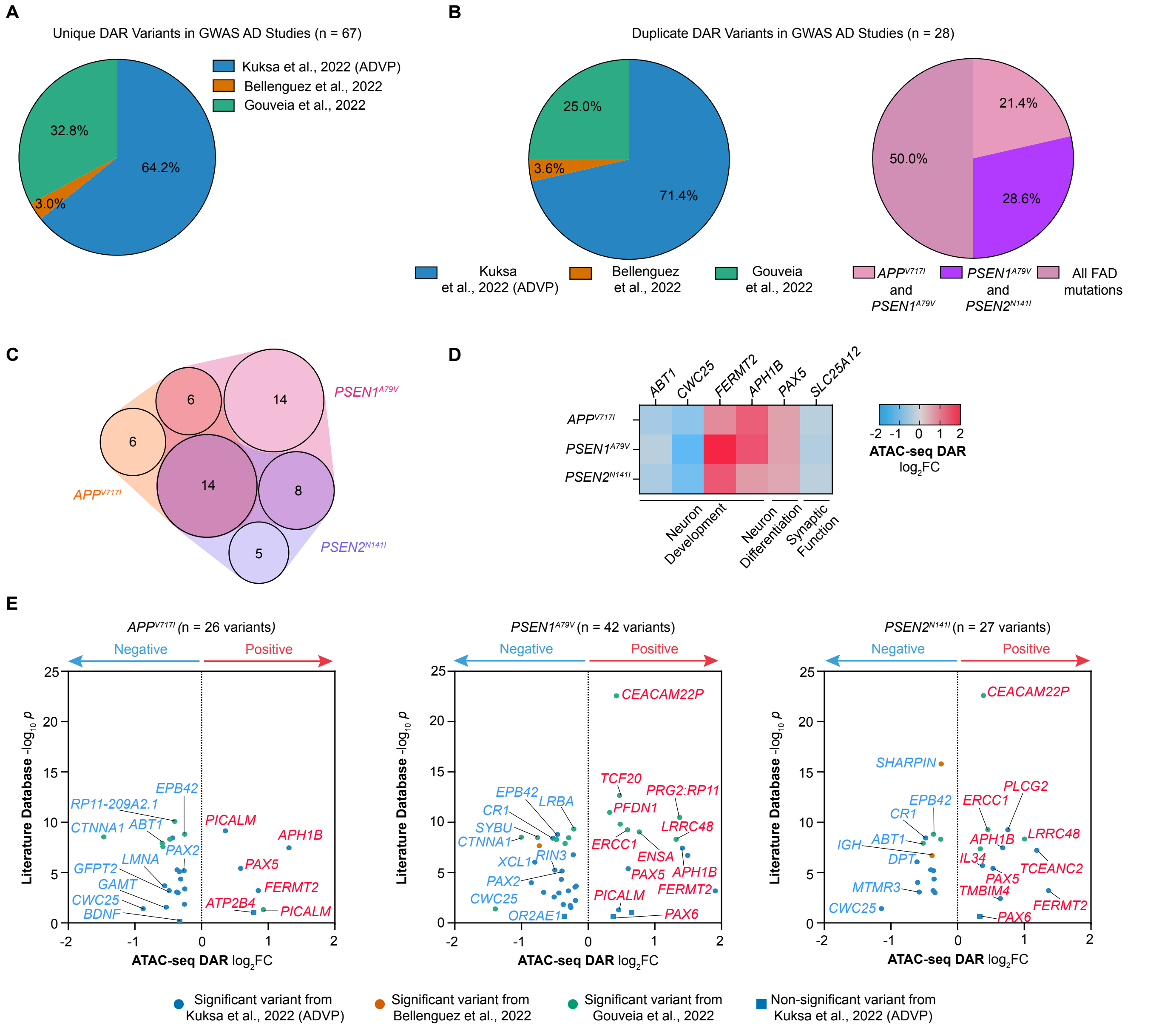

Supplementary Figure 9 *Profiling of ATAC-seq differentially accessible regions (DARs) in known AD related GWAS loci*

A Distribution of unique AD variants found in DARs based on published literature sources **B** Distribution of common AD variants found in DARs based on published literature sources (left) and FAD mutations (right). **C** Quasi-proportional Venn diagram overlap of AD variants found in DARs across the three FAD mutant hiPSC-derived neurons. **D** Subset of common AD variants across the FAD mutations with ATAC-seq DAR normalized log_2FC represented. **E** Curation of reported association p-values (log_{10} normalized; adjusted $p < 0.05$) across known literature sources for the AD variants found in DARs based against normalized log_2FC across the three FAD mutations (red, up-regulated variant; blue, down-regulated variant).

https://doi.org/10.1186/s13195-024-01659-6

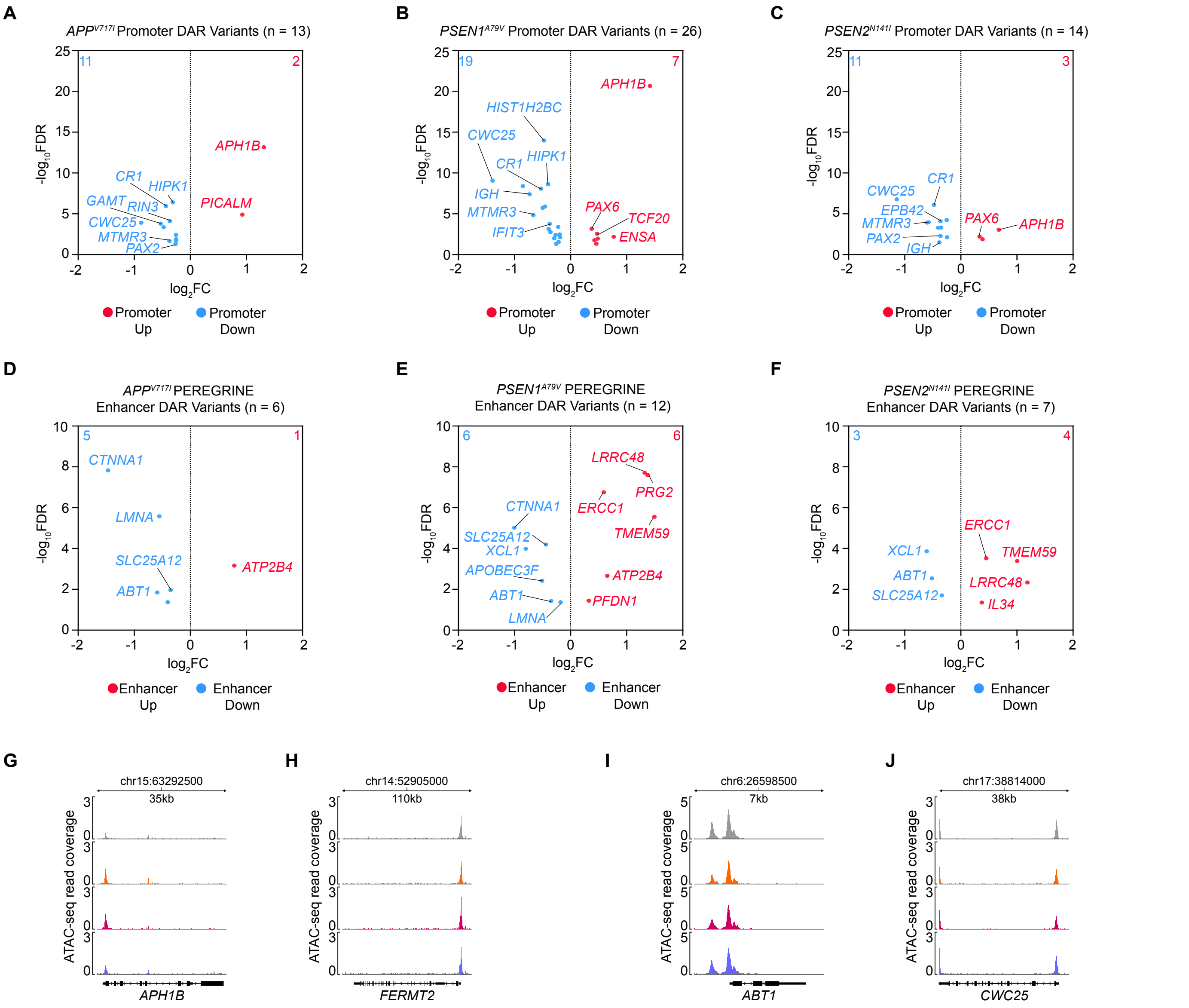

Supplementary Figure 10 *AD variants associated with ATAC-seq differentially accessible regions (DARs) in promoter associated and PEREGRINE enhancer associated regions*
A-C AD-associated variants (FDR *p*-value < 0.05) in with differential accessibility in promoter-associated regions showing increased or decreased accessibility across the three FAD mutations (APP^{V717I} vs. NDC in **A**, $PSEN1^{A79V}$ vs. NDC in **B**, and $PSEN2^{N141I}$ vs. NDC in **C**). **D-F** AD-associated variants (FDR *p*-value < 0.05) with differential accessibility in PEREGRINE enhancer-associated regions, showing increased or decreased accessibility across the three FAD mutations (APP^{V717I} vs. NDC in **D**, $PSEN1^{A79V}$ vs. NDC in **E**, and $PSEN2^{N141I}$ vs. NDC in **F**). **G-J** ATAC-seq coverage plots showing differential ATAC-seq peaks common across FAD mutant hiPSC-derived neurons occurring near AD GWAS variants with **G, H** increased accessibility or **I, J** decreased accessibility.

https://doi.org/10.1186/s13195-024-01659-6

A

Common Enriched Biological Pathways
with CADRO Mechanism Classes across all FAD mutations

Gene Ontological Biological Pathways	Common Alzheimer's Disease Research Onotology Classes
Biological Adhesion	Proteostasis/proteinopathies
Cell Fate Commitment	Neurogenesis
CNS Neuron Differentiation	
Neuron Fate Commitment	
Neuron Fate Specification	
Reg. of Neuron Differentiation	
Cerebral Cortex GABAeric Diff.	Synaptic plasticity/neuroprotection
G PRotein Coupled Receptor Sig.	Neurotransmitter receptors
Nervous System Process	Gut-brain axis
Taxis	Inflammation

B

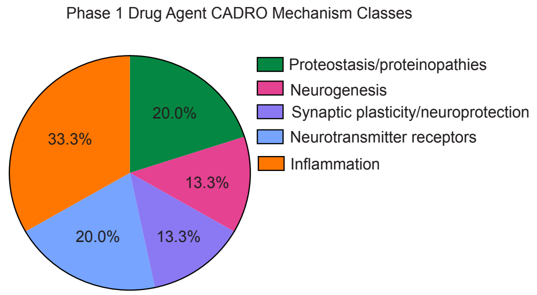

C

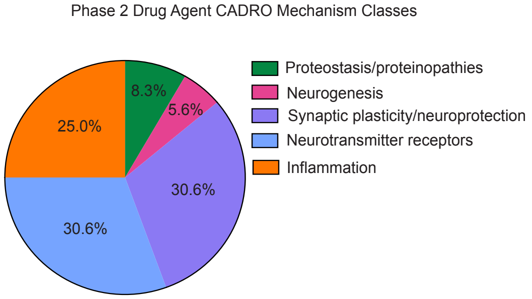

D

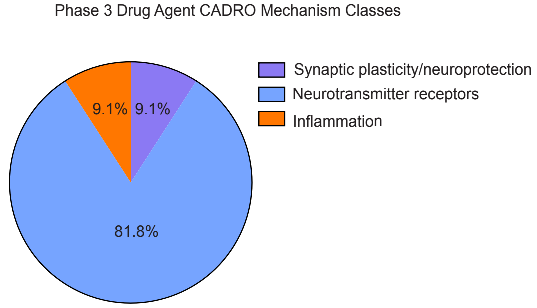

Supplementary Figure 11 *Characterization of integrated pathway-based drug agents associated with Common Alzheimer's disease Research Ontology (CADRO) mechanism classes*
A Table of commonly enriched integrated *intePareto* ranked biological pathways associated with CADRO-based mechanism classes across all FAD mutations. **B** Distribution of Phase 1 drug agents based on CADRO mechanism classes found in all FAD mutations. **C** Distribution of Phase 2 drug agents based on CADRO mechanism clases found in all FAD mutations. **D** Distribution of Phase 3 drug agents based on CADRO mechanism clases found in all FAD.

https://doi.org/10.1186/s13195-024-01659-6

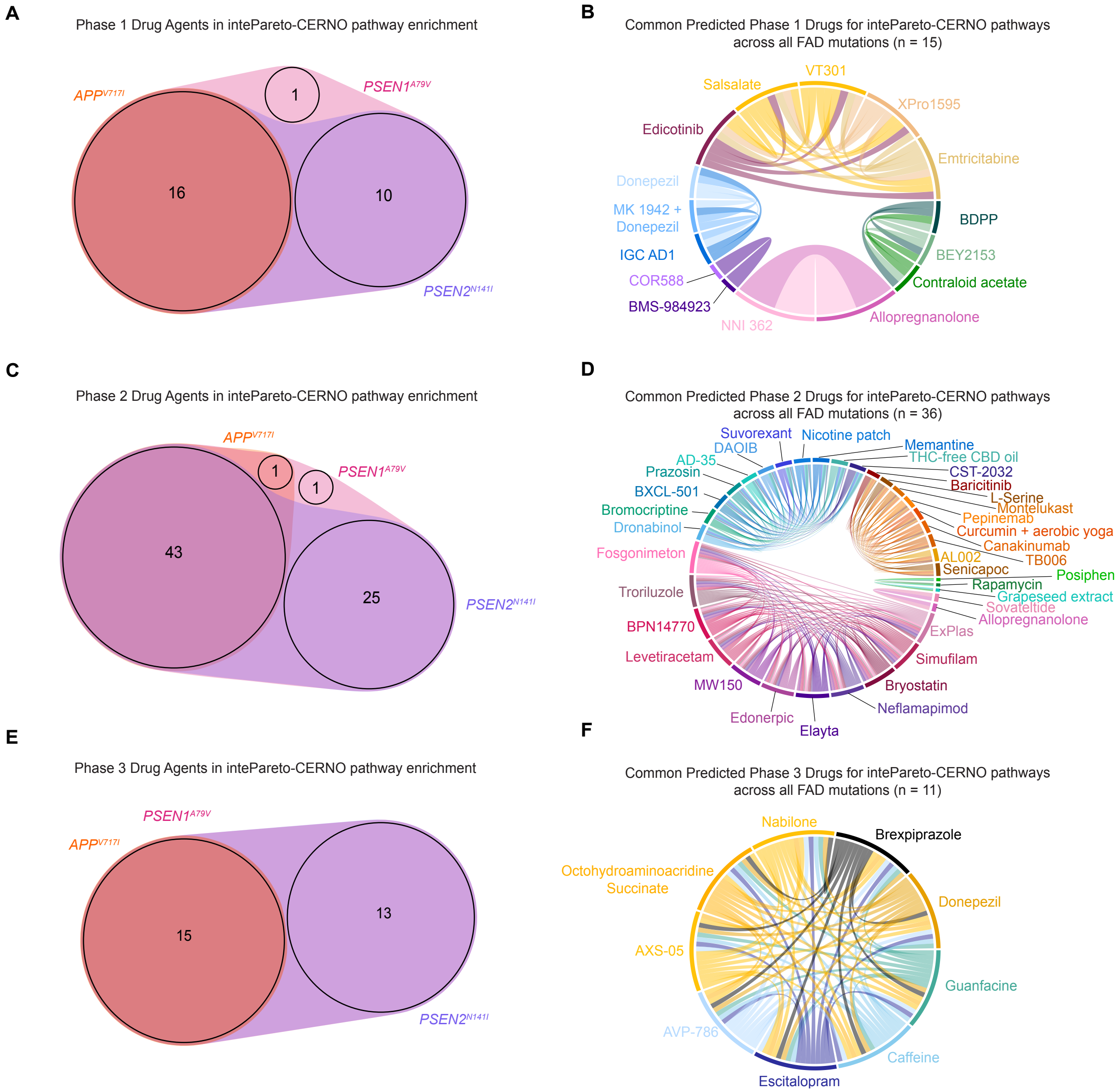

Supplementary Figure 12 *Profiling of drugs in drug trial phases in FAD hiPSC-derived neurons*
A Quasi-proportional Venn diagram overlap of integrated pathways across the three FAD mutant hiPSC-derived neurons of drug trial agents in Phase 1. **B** Chord diagram of predicted Phase 1 drug trial agents based on overlapping pathways from integration analysis across all FAD neurons. **C** Quasi-proportional Venn diagram overlap of integrated pathways across the three FAD mutant hiPSC-derived neurons of drug trial agents in Phase 2. **D** Circos plot of predicted Phase 2 drug trial agents based on overlapping pathways from integration analysis across all FAD neurons. **E** Quasi-proportional Venn diagram overlap of integrated pathways across the three FAD mutant hiPSC-derived neurons of drug trial agents in Phase 3. **F** Chord diagram of predicted Phase 3 drug trial agents based on overlapping pathways from integration analysis across all FAD neurons. Drug agent candidates related to proteostasis, neurogenesis, synaptic plasticity/neuroprotection, neurotransmitter receptors and inflammation in shades of green, pink, purple, blue/teal/yellow/black and red/orange/brown are shown, respectively.

https://doi.org/10.1186/s13195-024-01659-6

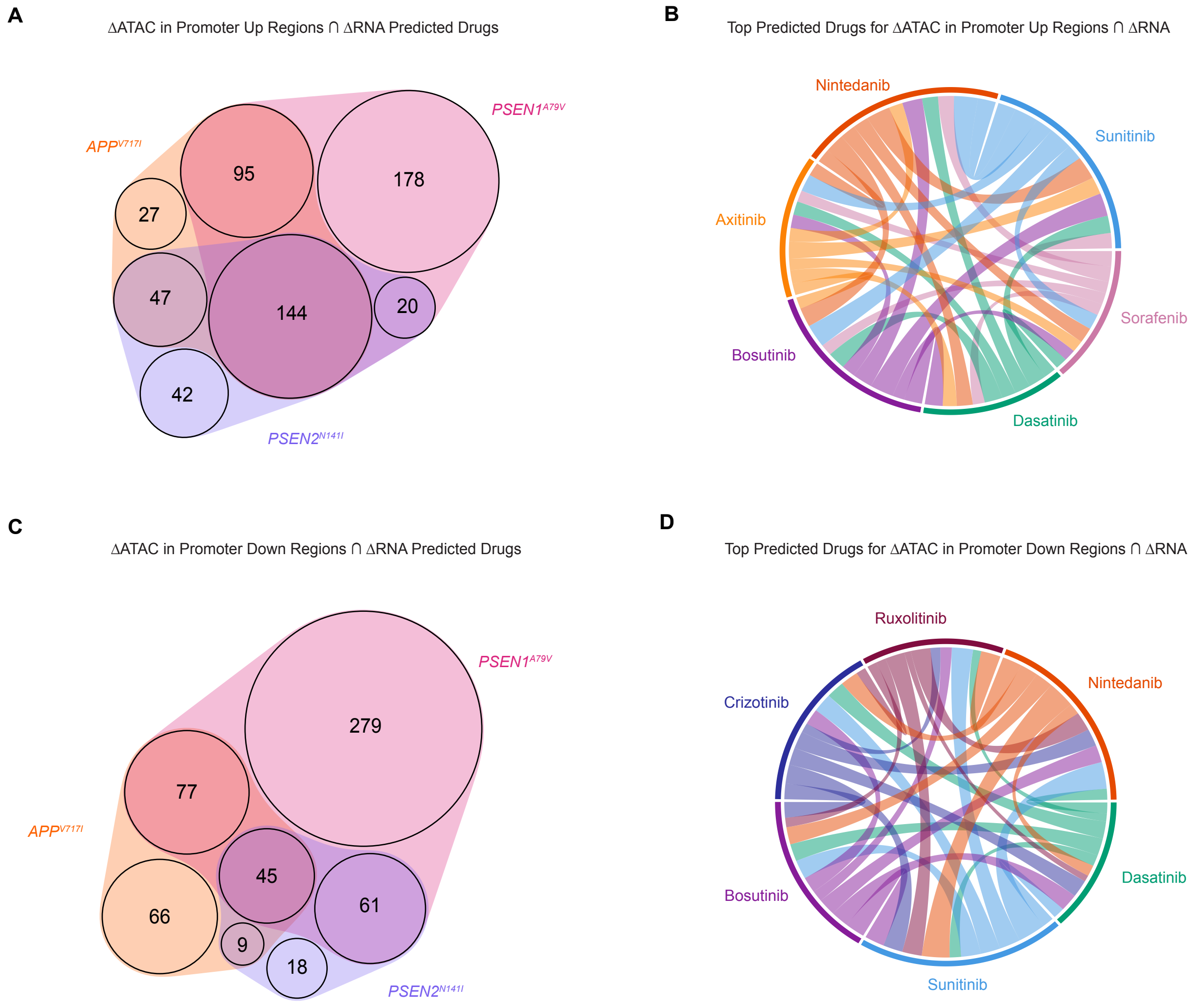

Supplementary Figure 13 *Profiling of predicted drug targets in FAD hiPSC-derived neurons in promoter regions with increased or decreased accessibility*

A Quasi-proportional Venn diagram overlap of drug targets across the three FAD mutant hiPSC-derived neurons in promoter-associated regions with increased accessibility. **B** Chord diagram of top predicted drug targets based on overlapping DEGs across all FAD neurons in promoter-associated regions with increased accessibility. **C** Quasi-proportional Venn diagram overlap of drug targets across the three FAD mutant hiPSC-derived neurons in promoter-associated regions with decreased accessibility. **D** Chord diagram of top predicted drug targets based on overlapping DEGs across all FAD neurons in promoter-associated regions with decreased accessibility.

https://doi.org/10.1186/s13195-024-01659-6

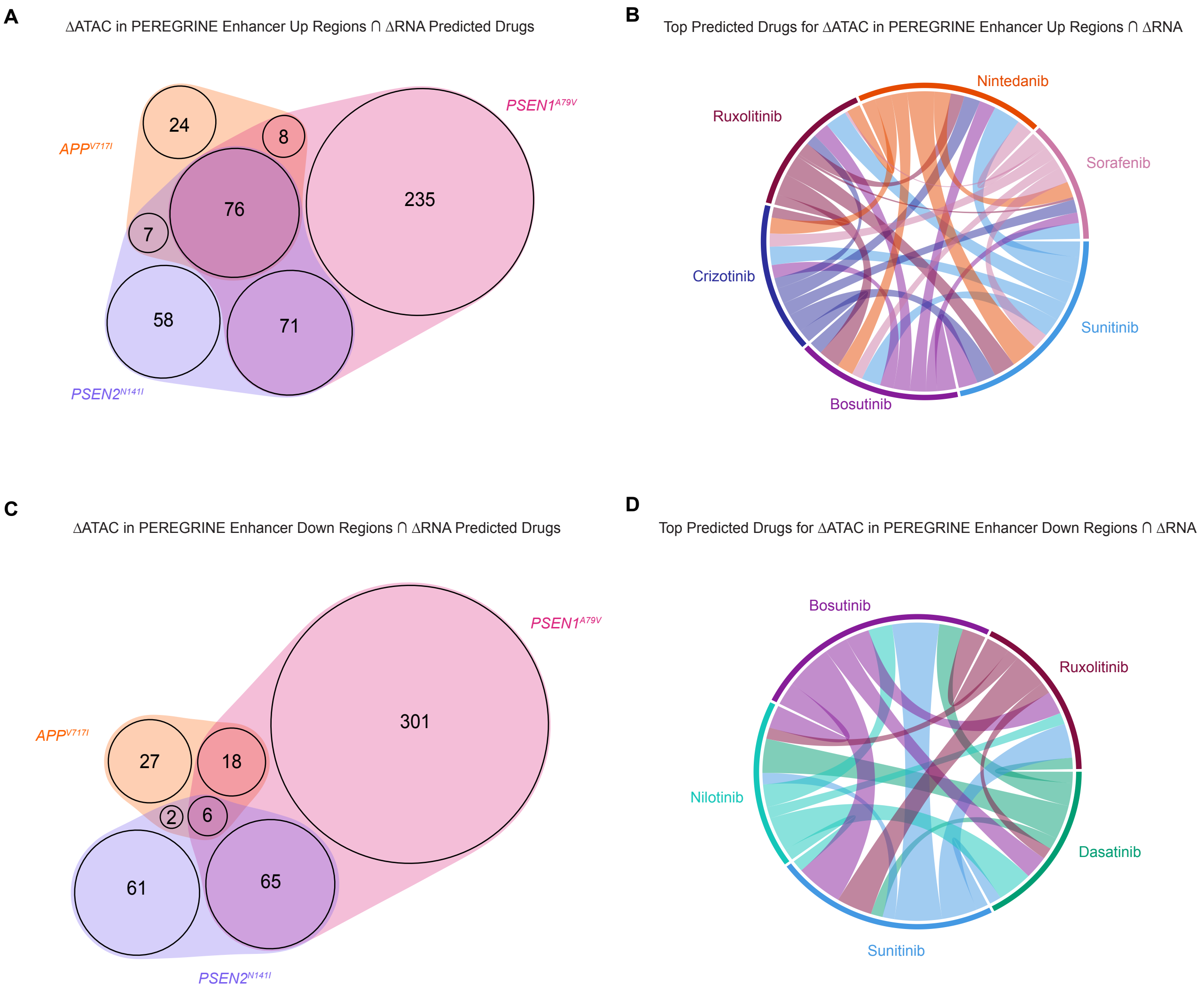

Supplementary Figure 14 *Profiling of predicted drug targets in FAD hiPSC-derived neurons in PEREGRINE-enhancer regions with increased or decreased accessibility*
A Quasi-proportional Venn diagram overlap of drug targets across the three FAD mutant hiPSC-derived neurons in PEREGRINE-enhancer regions with increased accessibility. **B** Chord diagram of top predicted drug targets based on overlapping DEGs across all FAD neurons in promoter-associated regions with increased accessibility. **C** Quasi-proportional Venn diagram overlap of drug targets across the three FAD mutant hiPSC-derived neurons in PEREGRINE-enhancer regions with decreased accessibility. **D** Chord diagram of top predicted drug targets based on overlapping DEGs across all FAD neurons in promoter-associated regions with decreased accessibility.

https://doi.org/10.1186/s13195-024-01659-6

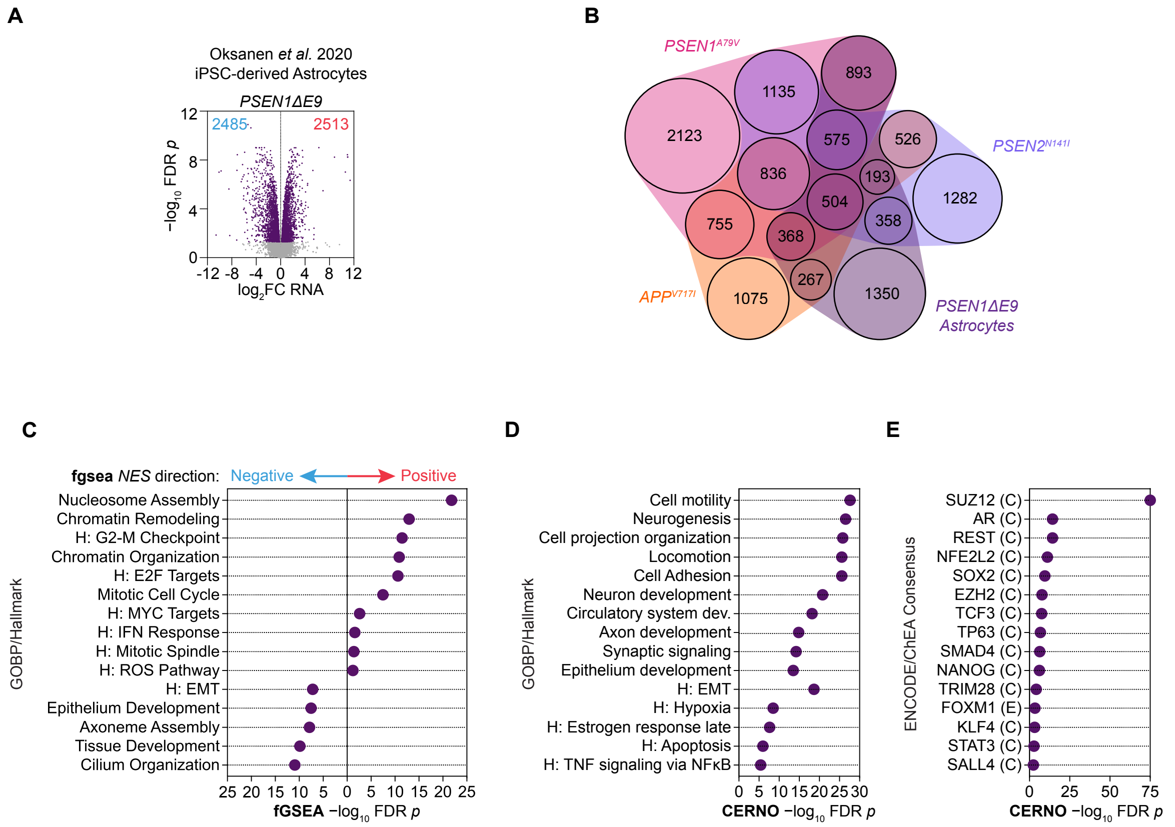

Supplementary Figure 15 *RNA-seq analysis of PSEN1ΔE9 hiPSC-derived astrocytes shows common pathway dysregulation as FAD iPSC-derived neurons*

A RNA-Seq volcano plot of differentially expressed genes (DEGs) in *PSEN1ΔE9* hiPSC-derived astrocytes as determined by *limma* with an FDR *p*-value < 0.05. **B** Quasi-proportional Venn diagram overlap of DEGs between *PSEN1ΔE9* astrocytes and *APP*^{V717I}, *PSEN1*^{A79V}, and *PSEN2*^{N141I} neurons. **C-D** Gene Ontology: Biological Process (GOBP) and Hallmark database geneset enrichment using the **C** *fgsea* multilevel enrichment test (left) or **D** *tmod* CERNO enrichment test (right). **E** ENCODE/ChEA Consensus TF-gene target database geneset enrichment using the *tmod* CERNO enrichment test. Dot plots indicate significant ($-\log_{10}$ FDR *p*-value < 0.05) pathways in each mutation relative to NDC.
